# Structural Insights into the Substrate Recognition of Ginsenoside Glycosyltransferase Pq3‐O‐UGT2

**DOI:** 10.1002/advs.202413185

**Published:** 2025-01-29

**Authors:** Qiushuang Ji, Yirong Liu, Huanyu Zhang, Yan Gao, Yixin Ding, Yuanyuan Ding, Jing Xie, Jianyu Zhang, Xinghua Jin, Bin Lai, Cheng Chen, Juan Wang, Wenyuan Gao, Kunrong Mei

**Affiliations:** ^1^ School of Pharmaceutical Science and Technology Faculty of Medicine Tianjin University Tianjin 300072 China; ^2^ Department of Pharmacology Hebei Medical University Shijiazhuang Hebei 050017 China; ^3^ Instrument Analytical Center School of Pharmaceutical Science and Technology Faculty of Medicine Tianjin University Tianjin 300072 China; ^4^ BMBF junior research group Biophotovoltaics Department of Microbial Biotechnology Helmholtz Centre for Environmental Research – UFZ 04318 Leipzig Germany; ^5^ School of Life Sciences Tianjin University Tianjin 300072 China; ^6^ State Key Laboratory for Quality Ensurance and Sustainable Use of Dao‐di Herbs Beijng 100700 China; ^7^ State Key Laboratory of Synthetic Biology Tianjin University Tianjin 300072 China

**Keywords:** enzymatic engineering, glycosyltransferase, regiospecificity, selectivity, substrate recognition

## Abstract

Ginsenosides are a group of tetracyclic triterpenoids with promising health benefits, consisting of ginseng aglycone attached to various glycans. Pq3‐O‐UGT2, an important UDP‐dependent glycosyltransferase (UGT), catalyzes the production of Ginsenoside Rg3 and Rd by extending the glycan chain of Ginsenoside Rh2 and F2, respectively, with higher selectivity for F2. However, the mechanism underlying its substrate recognition remains unclear. In this study, the crystal structures of Pq3‐O‐UGT2 in complex with its acceptor substrates are solved. The structures revealed a Nα5‐oriented acceptor binding pocket in Pq3‐O‐UGT2, shaped by the unique conformation of the Nα5‐Nα6 linker. Hydrophobic interactions play a pivotal role in the recognition of both Rh2 and F2, while hydrogen bonds specifically aid in F2 recognition due to its additional glucose moiety. The hydrophobic nature of the acceptor binding pocket also enables Pq3‐O‐UGT2 to recognize flavonoids. Overall, this study provides novel insights into the substrate recognition mechanisms of ginsenoside UGTs, advancing the understanding of their function and specificity.

## Graphical abstract

This work reveals the crystal structures of Pq3‐O‐UGT2 with its substrates (Rh2 or F2), unveiling an Nα5‐oriented binding pocket and unique linker conformation. Hydrophobic interactions are crucial for substrate recognition, while hydrogen bonds enhance specificity for F2. This study enhances the understanding of ginsenoside UGTs' substrate recognition mechanisms.

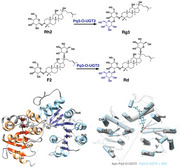

## 1 Introduction

Ginsenosides, a group of tetracyclic triterpenoid saponins, are the primary active ingredients of *Panax Ginseng*, a well‐known and highly valuable medicinal and dietary plant.^[^ ^1^, ^2^ ^]^ Dammarane‐type ginsenosides, which account for a significant portion of ginsenosides, comprise a protopanaxadiol (PPD) or protopanaxatriol aglycone and variable glycans.^[^ ^3^ ^]^ The glycans are attached to the C3‐OH and C20‐OH of PPD and the C3‐OH, C12‐OH, and C20‐OH of protopanaxatriol, respectively.

*Panax Ginseng* ingredients exhibit various pharmacological effects, such as anti‐tumor activity, immunity regulation, cardiovascular and cerebrovascular protection, anti‐aging properties, and memory improvement.^[^ ^4^, ^5^, ^6^ ^]^ These bioactivities are primarily attributed to rare ginsenosides, such as Ginsenoside CK, Rh2, Rg3, and F1.^[^ ^7^, ^8^ ^]^ Despite their versatile bioactivities, the content of rare ginsenosides in ginseng plants is extremely low.^[^ ^9^ ^]^ Currently, rare ginsenosides are primarily produced through the deglycosylation of ginsenosides,^[^ ^10^, ^11^ ^]^ which requires long cultivation periods for high‐quality ginseng roots and involves complex and costly extraction and purification processes.

**Figure 1:**
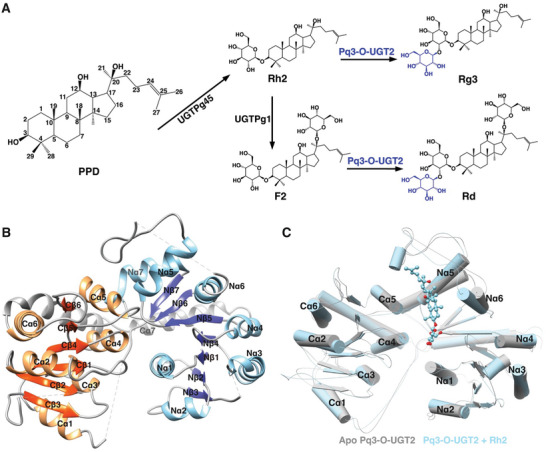
The function and overall structure of Pq3‐O‐UGT2. A) Enzymes involved in the biosynthesis of rare ginsenosides. The reactions catalyzed by Pq3‐O‐UGT2 are highlighted in blue. Pq3‐O‐UGT2 facilitates the glycosylation of Rh2 and F2, resulting in the formation of Rg3 and Rd, receptively. B) Crystal structure of apo Pq3‐O‐UGT2. The α helices and β strands in the N‐terminal domain are depicted in light and dark blue, respectively. The α helices and β strands in the C‐terminal domain are shown in orange and tan, respectively. Loops and the C‐terminal helix (Cα7) are presented in gray. C) Alignment of apo Pq3‐O‐UGT2 and the complex structure of Pq3‐O‐UGT2 with Rh2. Rh2 is represented as a ball‐and‐stick model, with oxygen atoms colored in red.

To increase the production of rare ginsenosides, significant efforts have been devoted to identifying their biosynthetic pathways in plants, which involve many UGTs as key enzymes^[^ ^12^ ^]^ (**Figure** **1**A). For example, Pq3‐O‐UGT1,^[^ ^13^ ^]^ PgUGT74AE2,^[^ ^14^ ^]^ and UGTPg45^[^ ^15^ ^]^ catalyze the glycosylation of the C3‐OH of PPD to form Rh2. Similarly, Pq3‐O‐UGT2,^[^ ^16^ ^]^ PgUGT94Q2,^[^ ^14^, ^17^ ^]^ and UGTPg29^[^ ^15^ ^]^ extend the glycan chain of Rh2 to produce Rg3. However, due to the limited catalytic efficiency and poor substrate selectivity of UGTs, the yields of rare ginsenosides remain considerably low. Efforts to improve the catalytic activity of plant UGTs toward ginsenosides have achieved limited success,^[^ ^18^, ^19^ ^]^ primarily constrained by the lack of structural information on these UGTs.

Plant UGTs exhibit low similarity in their sequence identities despite sharing a conserved GT‐B fold, which consists of two β/α/β Rossmann‐like structural domains. This feature enables them to catalyze a wide range of diverse substrates. To date, crystal structures of ≈40 plant UGTs have been reported.^[^ ^20^, ^21^, ^22^, ^23^, ^24^ ^]^ Most of these UGTs primarily recognize low molecule weight aglycones, such as simple phenols (*Arabidopsis thaliana* UGT72B1,^[^ ^25^ ^]^ *Persicaria tinctoria* UGT1,^[^ ^26^ ^]^ *Arabidopsis thaliana* UGT74F2^[^ ^27^ ^]^), trichothecene (*Oryza sativa* 79^[^ ^28^, ^29^ ^]^), and flavonoids (*Arabidopsis thaliana* UGT89C1,^[^ ^30^ ^]^ *Clitoria ternatea* UGT78K6,^[^ ^31^, ^32^ ^]^ *Glycyrrhiza glabra* CGT,^[^ ^33^ ^]^ *Mangifera indica* CGT,^[^ ^34^, ^35^ ^]^ *Landoltia punctata* CGTa,^[^ ^36^ ^]^ LpCGTb,^[^ ^36^ ^]^ *Scutellaria baicalensis* CGTa,^[^ ^36^ ^]^ SbCGTb,^[^ ^36^ ^]^ *Phytolacca americana* GT2,^[^ ^37^ ^]^ PaGT3,^[^ ^38^, ^39^ ^]^ *Vitis vinifera* GT1,^[^ ^40^ ^]^ *Zea mays* CGTa,^[^ ^36^ ^]^ *Glycyrrhiza uralensis* UGT79B74,^[^ ^22^ ^]^ *Scutellaria baicalensis* 3GT1,^[^ ^22^ ^]^ *Belamcanda chinensis* 7OUGT,^[^ ^21^ ^]^ and *Nemophila menziesii* F4′G7GT^[^ ^23^ ^]^). UGTs that recognize larger substrates include *Oryza sativa* UGT91C1^[^ ^41^ ^]^ and *Stevia rebaudiana* UGT76G1,^[^ ^42^, ^43^, ^44^ ^]^ which act on steviol glycosides, as well as *Calotropis gigantea* UGT74AN2^[^ ^45^ ^]^ and *Catharanthus roseus* UGT74AN3,^[^ ^24^ ^]^ which act on steroids. Other examples include *Siraitia grosvenorii* UGT74AC1,^[^ ^46^ ^]^ which processes mogrol, and the recently reported *Siraitia grosvenorii* UGT94‐289‐3,^[^ ^47^ ^]^ which glycosylates mogrosides. However, structural information on ginsenoside UGTs remains unavailable.

Although the substrates of UGT74AC1 are structurally similar to ginsenosides, engineering Pq3‐O‐UGT2 based on its predicted structure using UGT74AC1 as a template resulted in only a 1.3‐fold improvement in catalytic efficiency,^[^ ^19^ ^]^ suggesting different substrate recognition mechanisms between them. Thus, determining the structures of ginsenoside UGTs and their complexes with acceptors is crucial for understanding their substrate recognition mechanisms and optimizing them to enhance catalytic efficiency and specificity.

In this study, we elucidated the crystal structures of both apo Pq3‐O‐UGT2 and its complexes with Ginsenoside Rh2 and F2, revealing the acceptor recognition mechanisms of Pq3‐O‐UGT2. Structural analyses, combined with enzymatic assays, uncovered the molecular basis of its catalytic regiospecificity and guided the modification of the enzyme to enhance catalytic performance and achieve higher selectivity toward Rh2 over F2. This mutant enzyme holds promise as a biocatalyst for boosting Ginsenoside Rg3 production. Moreover, the structural insights from our study also shed light on substrate recognition in other UGTs that act on large substrates.

## 2 Results

### 2.1 Structure of Pq3‐O‐UGT2

Pq3‐O‐UGT2 catalyzes glucose transfer from UDP‐Glucose (UDP‐Glc) to the C2‐OH of the C3‐glucose of Ginsenoside Rh2 and F2, respectively (Figure 1A). To elucidate the underlying molecular mechanisms, we solved crystal structures of apo Pq3‐O‐UGT2 and its complex with Rh2 at resolutions of 2.9 and 3.5 Å, respectively (Table S1, Supporting Information). The apo structure of Pq3‐O‐UGT2 was determined via molecular replacement, utilizing *Phytolacca americana* GT2 (PaGT2, PDB ID: 6JEM) as a search model. The apo structure adopted a *P*1 space group with four molecules in the asymmetric unit, while the Pq3‐O‐UGT2‐Rh2 complex exhibited a *P*2_1_2_1_2_1_ space group with two molecules in the asymmetric unit (Figure S1A,B, Supporting Information). PISA^[^ ^48^ ^]^ analysis indicated the absence of higher‐order assemblies in solution, consistent with the monomeric state observed via gel filtration.^[^ ^49^ ^]^

Pq3‐O‐UGT2 features the conserved GT‐B fold, consisting of two Rossmann‐like domains: the N‐terminal domain (NTD, a.a. 1–222) and the C‐terminal domain (CTD, a.a. 239–442), connected by an intermediate loop (a.a. 223–238) (Figure 1B; Figure S2, Supporting Information). The NTD contains a seven‐stranded parallel β‐sheet sandwiched by seven helices, while the CTD comprises a 6‐stranded parallel β‐sheet flanked by 6 helices. The C‐terminal helix (Cα7) extends from the CTD to the NTD, stabilizing the two‐lobe structure of Pq3‐O‐UGT2.

In the apo structure, four regions are missing, including partial segments of the loop Nβ3‐Nα3 (a.a. 66–90), Nα5‐Nα6 (a.a. 168–177), Cβ2‐Cβ3 (a.a. 289–302), and the intermediate loop (a.a. 228–235). Alignment of the AlphaFold2^[^ ^50^ ^]^ predicted Pq3‐O‐UGT2 structure with each of the four molecules in the asymmetric unit suggests that the absence of density in Nβ3‐Nα3, Nα5‐Nα6, and Cβ2‐Cα3 is likely due to spatial conflict during crystal packing, while the missing density of the intermediate loop may result from flexibility (Figure S3A, Supporting Information). Compared to the apo structure, the structure of Pq3‐O‐UGT2 with Rh2 was better resolved, particularly in the Nα5‐Nα6 and Cβ2‐Cβ3 loops (Figures S2 and S3B, Supporting Information), indicating reduced spatial conflict during crystal packing in the different space group. Alignment of the two protomers in the complex structure of Pq3‐O‐UGT2 with Rh2 reveals slight variations in their loop regions (Figure S1D, Supporting Information), in contrast to the four identical protomers observed in the apo structure (Figure S1C, Supporting Information). These differences suggest that the loops, primarily located at the periphery of the structure, may undergo minor conformational changes to accommodate Rh2 binding.

In the complex structure, the density of Rh2 is clearly observed in the NTD of Pq3‐O‐UGT2 (Figure S1E,F, Supporting Information). However, the electron density of the alkyl tail is not well‐defined, likely due to its flexibility. Comparison of the apo and complex structures reveals significant conformational changes in the NTD (Figure 1C). In the complex structure, the NTD, particularly the Nα5, Nα6, and the Nα5‐Nα6 loop shift inward, with the most substantial movement occurring at the N‐terminus of Nα6 (≈5 Å). This indicates that Pq3‐O‐UGT2 undergoes conformational changes during substrate binding, and the binding of Rh2 induces a more compact conformation.

### 2.2 Catalytic Mechanism of Pq3‐O‐UGT2

**Figure 2:**
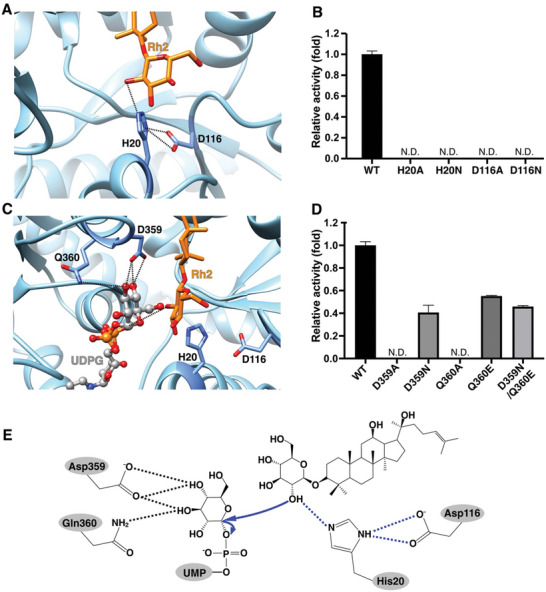
Catalytic mechanism of Pq3‐O‐UGT2. A) The His20‐Asp116 catalytic dyad of Pq3‐O‐UGT2. Hydrogen bonds are represented as dashed lines. B) Mutations of His20 and Asp116 deactivated Pq3‐O‐UGT2. N.D., not detectable. C) Asp359 and Gln360 form hydrogen bonds with UDP‐Glc. UDP‐Glc was docked into the Pq3‐O‐UGT2 and Rh2 complex structure with AutoDock Vina. Hydrogen bonds are represented as dashed lines. UDP‐Glc and Rh2 are represented as a gray ball‐and‐stick model and an orange stick model, respectively. Oxygen and nitrogen atoms are colored in red and blue, respectively. Phosphate atoms in UDP‐Glc are shown in orange. UDPG, UDP‐Glc. D) Mutations of Asp359 and Gln360 deactivated Pq3‐O‐UGT2 or decreased its catalytic activity toward Rh2. Error bars represent the standard deviation from 3 repeats. N.D.: not detectable. E) Schematic diagram illustrating the SN2‐like direct displacement catalytic mechanism of Pq3‐O‐UGT2. The His20‐Asp116 catalytic dyad deprotonates C2‐OH of the glucose moiety of Rh2, which subsequently undergoes nucleophilic attack on the anomeric carbon of UDP‐Glc, ultimately forming the inverted glycoside products.

Most plant O‐glycosyltransferases employ an SN2‐like direct replacement mechanism to catalyze glycosyl transfer from the sugar donor to the acceptor, wherein a Histidine‐Aspartate dyad acts as a general base to deprotonate the acceptor.^[^ ^20^ ^]^ In the complex structure of Pq3‐O‐UGT2, the His20 residue forms a hydrogen bond with the 2‐OH group of the C3‐glucose of Rh2, while Asp116 forms bidentate hydrogen bonds with His20 (**Figure** **2**A; Figure S4A, Supporting Information). Thus, His20 and Asp116 are proposed to form a catalytic dyad that deprotonates the C2‐OH group of Rh2. Substitutions of His20 or Asp116 with alanine or asparagine completely abolished enzymatic activity (Figure 2B), confirming that the His20‐Asp116 dyad functions as the catalytic base deprotonating Rh2.

The docking of UDP‐Glc into the Pq3‐O‐UGT2‐Rh2 complex structure closely resembles the configuration of UDP‐Glc bound to other UGTs (Figure S5, Supporting Information). In this configuration, Gln360 establishes a hydrogen bond with the C3‐OH group of UDP‐Glc, while Asp359 forms bidentate hydrogen bonds with the C3‐ and C4‐OH groups (Figure 2C). Gln360 and Asp359 are the last two residues of the highly conserved plant secondary product glycosyltransferase (PSPG) motif, which plays key roles in sugar donor recognition.^[^ ^20^ ^]^ Mutagenesis experiments revealed that substituting Asp359 with asparagine or Gln360 with glutamate reduces the catalytic activity of Pq3‐O‐UGT2, while substituting either residue with alanine completely abolishes enzymatic activity (Figure 2D). These findings confirm that Asp359 and Gln360 are crucial for the O‐glycosylation function of Pq3‐O‐UGT2, as they stabilize the sugar moiety of the donor molecule, consistent with previous studies.^[^ ^25^, ^40^, ^51^ ^]^ Additionally, the anomeric carbon of UDP‐Glc is positioned in close proximity to the C2‐OH group of the C3‐glucose of Rh2 (Figure 2C), facilitating the nucleophilic attack from deprotonated Rh2 and subsequent glycosyl transfer. This completes the SN2‐like direct replacement mechanism catalyzed by Pq3‐O‐UGT2 (Figure 2E).

### 2.3 Pq3‐O‐UGT2 Binds to Ginsenoside Rh2 with an Nα5‐Oritented Pocket

**Figure 3:**
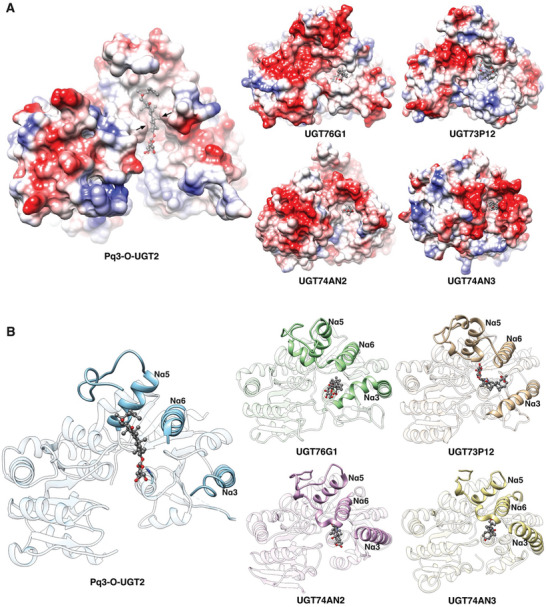
Pq3‐O‐UGT2 binds to Rh2 with an Nα5‐orientated pocket. A) The binding pockets of Pq3‐O‐UGT2, UGT76G1, UGT73P12, UGT74AN2, and UGT74AN3. Acceptors are represented as stick‐and‐ball models. The surface is colored according to the electrostatic potential using the Coulombic Surface Coloring method. The gateway cradling the tetracyclic ring of Rh2 is indicated by arrows. B) The binding pockets illustrated by ribbons. The Nα3, Nα5, Nα6 helices, and the Nα5‐Nα6 linker are emphasized in the complex structures, with other parts presented in semitransparency.

The Rh2‐binding pocket of Pq3‐O‐UGT2 is primarily formed by Nα5 (a.a. 141–152), Nα6 (a.a. 179–191), and Cα5 (a.a. 358–369), with the Nα5‐Nα6 linker (a.a. 153–178) acting as a lid that covers the tail of Rh2 (Figure 1C). The pocket is elongated, deep, and highly hydrophobic to accommodate the Rh2 molecule, which has a rigid, extended tetracyclic hydrophobic backbone and a flexible alkyl tail (Figures 1A and 3A). Meanwhile, the pocket's wide surface opening suggests a large entrance for acceptor molecules.

The acceptor binding pocket of Pq3‐O‐UGT2 differs significantly from those of other UGTs that catalyze large, extended acceptors, such as *Glycyrrhiza uralensis* UGT73P12, *Stevia rebaudiana* UGT76G1,^[^ ^42^, ^43^, ^44^ ^]^ *Calotropis gigantea* UGT74AN2,^[^ ^45^ ^]^ and *Catharanthus roseus* UGT74AN3.^[^ ^24^ ^]^ These UGTs possess narrow, deep tunnels with small surface openings in their acceptor binding pockets (**Figure** **3**A). Additionally, the acceptor binding pocket of Pq3‐O‐UGT2 extends toward the Nα5 helix, whereas the pockets of UGT73P12, UGT76G1, UGT74AN2, and UGT74AN3 are primarily formed along the Nα3 and Nα6 helices (Figure 3B; Figure S6A, Supporting Information). As a result, the orientation of the acceptor binding pocket in Pq3‐O‐UGT2 is nearly perpendicular to that found in many terpenoid and steroid UGTs (Figure S6B, Supporting Information).

Interestingly, in UGT76G1, UGT73P12, UGT74AN2, and UGT74AN3, the acceptors bind in a “headstand” orientation (referred to as Nα3 orientation). In this configuration, the glycosylated group (the head of the acceptor) faces downward toward the catalytic histidine residue, while the backbone of the aglycone rests against the Nα3 helix (Figure 3B; Figure S6A, Supporting Information). By contrast, in Pq3‐O‐UGT2, Rh2 adopts a “lying” orientation (referred to as Nα5 orientation), with its tail extending toward the Nα5 helix. These differences in binding orientation suggest two distinct mechanisms of acceptor entry into the binding pocket: in Pq3‐O‐UGT2, the head and the backbone of Rh2 may enter the pocket simultaneously, whereas in UGT73P12, UGT76G1, UGT74AN2, and UGT74AN3, the head of the acceptor enters first, followed by the backbone.

Interestingly, UGT91C1, which catalyzes the glycosylation of diterpenoids, also has an acceptor binding pocket that extends toward the Nα5 helix (Figure S7A, Supporting Information), similar to Pq3‐O‐UGT2. However, the upper portion of the pocket is absent in UGT91C1 (Figure S7A, Supporting Information), indicating greater flexibility in its Nα5‐Nα6 linker. Moreover, compared to UGT91C1, the acceptor binding pocket of Pq3‐O‐UGT2 is longer and deeper, enabling it to accommodate larger triterpenoid aglycones (Figure 3A; Figure S7B,C, Supporting Information).

### 2.4 The Conformation of the Nα5‐Nα6 Linker is Crucial for Acceptor Binding Pocket Formation

**Figure 4:**
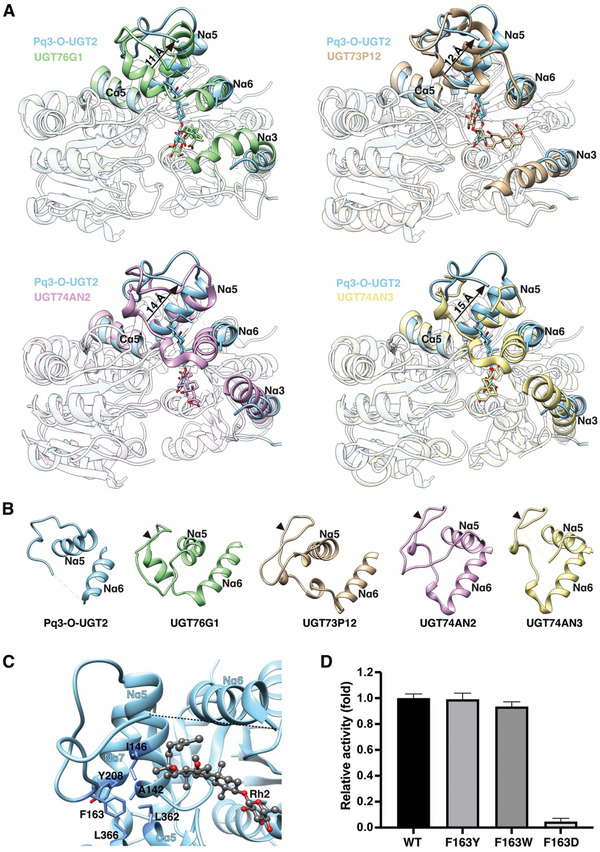
The conformation of the Nα5‐Nα6 linker is crucial for acceptor binding pocket formation. A) Structure alignment of Pq3‐O‐UGT2 and other UGTs catalyzing large and extended acceptors. Displacement of the Nα5‐Nα6 linker is indicated. B) The conformation of the Nα5‐Nα6 linker in different UGTs. The hairpin is indicated by arrow heads. C) The Nα5‐Nα6 linker of Pq3‐O‐UGT2 is stabilized by a hydrophobic center. D) Disruption of the hydrophobic interactions of Phe163 inhibited the catalytic activity of Pq3‐O‐UGT2 toward Rh2. Error bars represent standard deviation from 3 repeats.

The most notable distinction between Pq3‐O‐UGT2 and other UGTs lies in the linker connecting the Nα5 and Nα6 helices. In Pq3‐O‐UGT2, the Nα5‐Nα6 linker moves outward by 11–15 Å compared to UGT76G1, UGT73P12, UGT74AN2, and UGT74AN3 (**Figure** **4**A). Furthermore, in these other UGTs, the Nα5‐Nα6 linker features a hairpin structure (Figure 4B). This hairpin interacts with Nα5, Nα7, and Cα5, stabilizing the subsequent linker connecting to Nα6 and thereby restricting the accessibility of Nα5 to acceptor substrates. However, this hairpin structure is absent in Pq3‐O‐UGT2 (Figure 4B). In Pq3‐O‐UGT2, the N‐terminal portion of the Nα5‐Nα6 linker forms a simple turn stabilized by interactions with Nα5, Nα7, and Cα5, while the C‐terminal portion extends away from Cα5 to connect with Nα6 (Figure 4A; Figure S4B, Supporting Information), resulting in a broader opening at the top surface of the acceptor binding pocket.

Given the significant structural disparity in the Nα5‐Nα6 linker between Pq3‐O‐UGT2 and other UGTs, we propose that the conformation of the Nα5‐Nα6 linker is pivotal for the function of Pq3‐O‐UGT2. Phe163, located at the base of the turn of the Nα5‐Nα6 linker, plays a key role in stabilizing its conformation through hydrophobic interactions with Ala142 and Ile146 of Nα5, Tyr208 of Nα7, and Leu362 and Leu366 of Cα5 (Figure 4C). Notably, Phe163 does not directly interact with the substrate. Mutations of Phe163 to tyrosine or tryptophan, which preserve the hydrophobic interactions, result in minimal effects on the catalytic activity of Pq3‐O‐UGT2. In contrast, the substitution of Phe163 with aspartate, which disrupts the hydrophobic interactions, almost completely abolishes its catalytic activity toward Rh2 (Figure 4D). These results strongly support the hypothesis that the conformation of the Nα5‐Nα6 linker is crucial for the formation and function of the acceptor binding pocket in Pq3‐O‐UGT2.

### 2.5 Hydrophobic Interactions Play a Pivotal Role in Ginsenoside Rh2 Recognition

**Figure 5:**
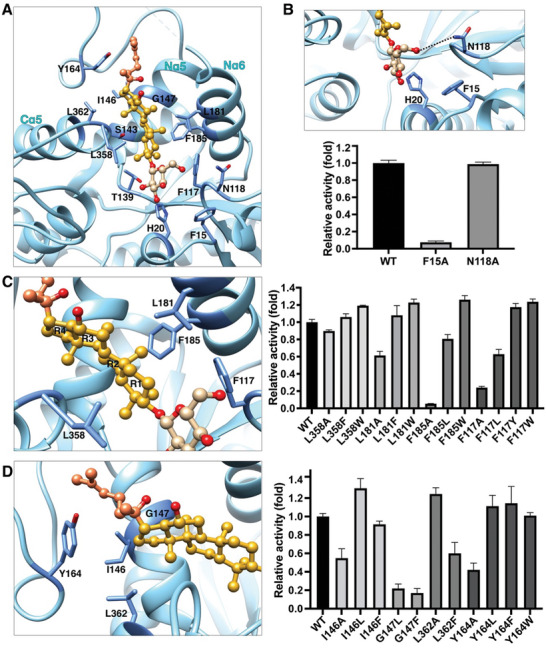
Hydrophobic interactions play a pivotal role in Rh2 recognition. A) The Rh2 binding pocket of Pq3‐O‐UGT2. Ginsenoside Rh2 is presented as a ball‐and‐stick model, with the glucose moiety colored in tan, the tetracyclic ring in gold, alky tail in coral, and oxygen atoms in red. Residues forming the Rh2 binding pocket are labeled and highlighted as sticks. B) Residues interacting with the glucose moiety of Rh2 and the relative activities of corresponding mutants toward Rh2. Hydrogen bonds are represented as dashed lines. C) Residues interacting with the tetracyclic ring of Rh2 and the relative activities of corresponding mutants toward Rh2. R1‐R4, Ring1‐ Ring4. D) Residues interacting with Ring 4 and the alky tail of Rh2 and the relative activities of corresponding mutants toward Rh2. Error bars represent standard deviation from 3 repeats.

The Rh2‐binding pocket of Pq3‐O‐UGT2 is composed of His20, Phe15, Phe117, Asn118, Thr139, Ser143, Ile146, Gly147, Tyr164, Leu181, Phe185, Leu358, and Leu362 (**Figure** **5**A; Figures S4C, S8, Supporting Information). Surrounding the head of Rh2, Asn118 forms a hydrogen bond with the C6‐OH group of the glucose moiety, while Phe15 engages in hydrophobic interactions with the glucose ring (Figure 5B). Mutation of Phe15 to alanine nearly abolishes the catalytic activity of Pq3‐O‐UGT2 toward Rh2, whereas the N118A mutation had no significant effect, suggesting that the hydrophobic interaction between Phe15 and Rh2 is crucial for substrate recognition.

In the central region of the Rh2‐binding pocket, the Nα6 helix and the N‐terminal tip of the Cα5 helix form a gateway that cradles the tetracyclic ring of Rh2 (Figure 3A and 5A). Leu181 and Phe185 from Nα6, along with Leu358 at the tip of Cα5, engage in hydrophobic interactions with Rings 1 and 2 of Rh2 (Figure 5C). Leu358 and Leu181 are positioned near the top surface of the pocket, while Phe185 resides deeper within. Additionally, Phe117 forms a hydrophobic interaction with Ring 1 of Rh2 and is located inside the pocket. These residues form a hydrophobic core with Rh2, stabilizing its backbone. Mutating Leu358 to alanine slightly reduces catalytic activity, whereas mutating it to tryptophan enhances activity (Figure 5C). Similarly, the L181A mutation decreases activity, while the L181W mutation increases it. These results suggest that larger hydrophobic side chains at these positions stabilize the substrate, though side‐chain size is not the sole determinant of activity. In contrast, the residue at position 185 has a strict size requirement: the F185A mutation nearly abolishes activity, the F185L mutation decreases it, and the F185W mutation increases activity. Mutations of Phe117 show similar size effects to those of Phe185 (Figure 5C). AlphaFold2‐predicted structures of Pq3‐O‐UGT2 mutants indicate that larger side chains (e.g., tryptophan) approach Rh2 more closely, while smaller side chains (e.g., alanine) create greater distance without altering the overall structure (Figure S9, Supporting Information). These results suggest a “space‐filling” effect on hydrophobic interactions, where larger hydrophobic residues enhance Rh2 recognition and binding. The size requirements at positions 185 and 117, which are deep within the pocket, further indicate that the central portion of the pocket is spacious but has limited capacity.

To explore whether additional filling of the binding pocket could enhance catalytic efficiency, L181W was combined with L358W or F185W to generate double mutations. However, both double mutations reduced catalytic activity (Figure S10A, Supporting Information). The L181W/F185W/L358W triple mutation resulted in a dramatic loss of activity. Structure prediction showed no significant changes in the overall structure compared to the wildtype (Figure S10B, Supporting Information), suggesting that reduced activity is due to alterations in the binding pocket rather than global structural changes. These findings indicate that, although the central pocket is spacious, its capacity for further modifications is limited.

At the rear of the pocket, Ile146, Gly147, Tyr164, and Leu362 form another hydrophobic center that interacts with Ring 4 and the alky tail of Rh2. Ile146, Gly147, and Leu362 are positioned at the bottom, while Tyr164 is near the top (Figure 5D). Mutation of Ile146 to alanine or phenylalanine reduces activity, whereas mutation to leucine, which has a similar side‐chain size, increases activity. This suggests that both hydrophobicity and side‐chain size at position 146 are critical for Rh2 interaction. Mutating Gly147 to leucine or phenylalanine significantly decreases catalytic activity, indicating that a side chain at this position is not favorable. In contrast, mutating Leu362 to alanine increases activity, while mutating it to phenylalanine decreases activity, suggesting that a small side chain at this position is optimal. These findings suggest that the rear bottom of the pocket fits Rh2 well, and introducing larger side chains may cause steric conflicts. Interestingly, mutating Tyr164 to leucine, phenylalanine, or tryptophan has minimal effects on activity, but mutation to alanine decreases activity, suggesting that hydrophobicity, rather than sidechain size, is critical at this position.

Two polar residues, Thr139 and Ser143, are located at the bottom of the Rh2‐binding pocket but do not form hydrogen bonds with Rh2 (Figure S11A, Supporting Information). Mutating them to alanine decreases activity (Figure S11B, Supporting Information), suggesting that their polarity is important for catalysis. Conversely, the T139S and S143T mutations enhance activity, indicating that subtle modifications to their side chains without disrupting polarity are favorable for catalysis. Notably, Ser143 is close to Asp359, which forms hydrogen bonds with the glucose moiety of UDP‐Glc (Figure 2C; Figure S11A, Supporting Information), suggesting that it may indirectly stabilize the sugar donor. Thr139 forms a direct hydrogen bond with the C6‐OH of UDP‐Glc in the docked model (Figure S11A, Supporting Information), consistent with the roles of corresponding residues in other UGTs.^[^ ^29^, ^32^ ^]^ Polar residues in the Nβ5‐Nα5 linker, located at the bottom of the binding pocket, are conserved across most resolved plant UGTs (Figure S11C, Supporting Information). Thus, Thr139 and Ser143 are likely involved in donor recognition rather than direct interaction with Rh2.

### 2.6 Both Hydrophobic Interactions and Hydrogen Bonds are Crucial for Ginsenoside F2 Recognition

In addition to Rh2, Ginsenoside F2 is another known substrate of Pq3‐O‐UGT2. F2 contains one mono‐glucose moiety attached to each end (C3 and C20) of its tetracyclic ring.^[^ ^16^ ^]^ Interestingly, Pq3‐O‐UGT2 exhibits high regiospecificity toward F2, exclusively catalyzing β(1‐2) glycosylation of the C3‐O‐glucose of F2 (Figure S12, Supporting Information). This is in contrast to many other UGTs, where hydrophobic substrate recognition often leads to substrate promiscuity and poor regiospecificity. For instance, UGT74AN2 possesses a hydrophobic acceptor binding pocket and catalyzes a wide range of structurally diverse compounds.^[^ ^45^ ^]^ Similarly, hydrophobic interactions allow UGT76G1 and UGT91C1 to catalyze multiple substrates in different orientations.^[^ ^41^, ^42^ ^]^

**Figure 6:**
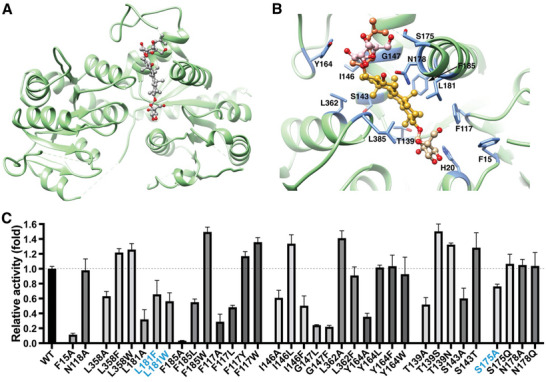
Recognition of F2 by Pq3‐O‐UGT2. A) Crystal structure of Pq3‐O‐UGT2 in complex with Ginsenoside F2. F2 is presented as a stick‐and‐ball model, with oxygen atoms shown in red. B) The F2 binding pocket of Pq3‐O‐UGT2. F2 is presented as a stick‐and‐ball model, with the C3‐glucose colored in tan, the tetracyclic ring in gold, alky tail in coral, C20‐glucose in pink, and oxygen atoms in red. Residues forming the F2 binding pocket are labeled and highlighted as sticks. C) Relative activities of Pq3‐O‐UGT2 mutants toward F2. Error bars represent standard deviation from 3 repeats. Mutations with different effects on the catalytic activity toward F2 and Rh2 are highlighted in blue.

To elucidate the mechanism of F2 recognition by Pq3‐O‐UGT2, we determined the crystal structure of the Pq3‐O‐UGT2‐F2 complex at a resolution of 3.4 Å (**Figure** **6**A; Table S1, Supporting Information). Like the Pq3‐O‐UGT2‐Rh2 complex, Pq3‐O‐UGT2‐F2 adopts a *P*2_1_2_1_2_1_ space group with 2 molecules in the asymmetric unit. These 2 molecules align well with each other, as well as with the apo Pq3‐O‐UGT2 and Pq3‐O‐UGT2‐Rh2 structures, despite some variations in loop regions (Figure S13A, Supporting Information). Similar to Rh2 binding, F2 binding induces inward movement of the Nα6 helix compared to the apo Pq3‐O‐UGT2 structure.

The electron density of F2 is clearly observed in the acceptor binding pocket of the NTD, with the 2 mono‐glucose moieties well assigned, although the partial density of the alky tail remains unresolved (Figure S13A,B, Supporting Information). The backbone of F2 aligns well with that of Rh2 (Figure S13A, Supporting Information), indicating that the hydrophobic interactions that govern Rh2 recognition also extend to F2. Indeed, residues crucial for Rh2 recognition also interact with F2 (Figures 5A and 6B). Mutations of these residues show similar effects on catalytic activities toward F2 as they do for Rh2 (Figures 5B–D and 6C), confirming the importance of hydrophobic interactions in F2 recognition. Interestingly, the “space‐filling” effect observed for Rh2 recognition at position 181 does not apply to F2 (Figure 6C). Mutations of Leu181 to alanine, phenylalanine, or tryptophan all decreased catalytic activity toward F2. This discrepancy may arise from the additional glucose moiety in F2, which causes a slight shift in the N‐terminal end of the Nα6 helix compared to Rh2 binding (Figure S13A, Supporting Information), indirectly affecting interactions between Leu181 and the aglycone backbone.

At the rear of the F2‐binding pocket, the C20‐O‐glucose moiety forms a hydrogen bond with Tyr164, which also interacts hydrophobically with the alky tail of F2 (Figure S14A, Supporting Information). However, mutation of Tyr164 to leucine, phenylalanine, or tryptophan does not affect catalytic activity, whereas mutation to alanine decreases activity (Figure 6C). These findings suggest that the hydrophobic interaction between Tyr164 and the alky tail is more crucial for F2 recognition than the hydrogen bond with the C20‐O‐glucose.

Additionally, the C20‐O‐glucose lies near the N‐terminal end of the Nα6 helix, which harbors two polar residues, Ser175 and Asn178, oriented toward the glucose moiety (Figure 6B; Figure S14A, Supporting Information). Although poor electron density prevents precise modeling of their side chains, their proximity suggests potential hydrogen bonding, either directly or via water molecules. Indeed, the S175A mutation decreases activity toward F2 by ≈30% (Figure 6C; Figure S14B, Supporting Information). In contrast, the N178A mutation does not affect activity. Importantly, neither the S175A nor N178A mutations impact catalytic activity toward Rh2 (Figure S14B, Supporting Information), suggesting that the hydrogen bond formed between Ser175 and the C20‐O‐glucose is specific to F2 recognition. Together, these data reveal that hydrophobic interactions, complemented by a specific hydrogen bond involving the C20‐O‐glucose of F2, contribute to the regiospecificity of Pq3‐O‐UGT2 toward F2.

### 2.7 A Pq3‐O‐UGT2 Mutant with Enhanced Catalytic Activity and Selectivity Toward Ginsenoside Rh2

The glycosylation product of Ginsenoside Rh2 is the rare Ginsenoside Rg3, a primary component of traditional Chinese anti‐tumor drugs,^[^ ^52^ ^]^ whereas that of Ginsenoside F2 is Ginsenoside Rd, which has lower pharmacological significance. Since the catalytic efficiency of wildtype Pq3‐O‐UGT2 toward F2 is ≈4‐fold higher than that toward Rh2 (**Table** **1**), engineering a Pq3‐O‐UGT2 mutant with enhanced catalytic activity and selectivity for Rh2 would significantly benefit the production of Ginsenoside Rg3.

**Table 1:** Apparent kinetic parameters of Pq3‐O‐UGT2 and it is representative mutants toward Rh2 and F2.

Mutant	Substrate	*K*_M_, µM	*k*_cat_, s^−1^	*k*_cat_/*K*_M_, s^−1^ mΜ^−1^	Fold to WT
Wild type	Rh2	130.02 ± 25.81	3.71 ± 0.31	28.54 ± 2.41	1
	F2	77.69 ± 8.46	9.16 ± 0.34	117.94 ± 4.34	1
L181W	Rh2	72.17 ± 10.49	6.51 ± 0.26	90.17 ± 3.61	3.15
	F2	83.61 ± 8.43	5.04 ± 0.23	60.29 ± 2.75	0.51
S175A	Rh2	90.83 ± 14.35	2.65 ± 0.14	29.19 ± 1.53	1.02
	F2	80.52 ± 12.23	5.01 ± 0.27	62.22 ± 3.40	0.53
S175A /L181W	Rh2	45.43 ± 3.61	5.74 ± 0.13	126.40 ± 2.93	4.43
	F2	99.98 ± 13.00	5.43 ± 0.21	54.29 ± 2.05	0.46

The standard deviations were calculated from three replicates.

Through analysis of acceptor recognition mechanisms, several Pq3‐O‐UGT2 mutants with increased catalytic activity toward Rh2 were identified (Figure 5C,D). The kinetic parameters of these mutants for Rh2 and F2 were subsequently measured (Figures S15 and S16, Table S2, Supporting Information). Consistent with their improved conversion rates, these mutants exhibited increased catalytic efficiency (*k*_cat_/*K*_M_) toward Rh2 compared to the wildtype enzyme, with most showing a 2‐fold enhancement. However, catalytic efficiency toward F2 showed only modest improvement, likely due to the already high efficiency of the wildtype enzyme toward F2. Remarkably, the L181W mutant demonstrated a significant increase in catalytic efficiency toward Rh2 while exhibiting reduced efficiency toward F2. The catalytic efficiency of L181W toward Rh2 (90.17 ± 3.61 s^−1^ mΜ^−1^) surpassed that of F2 (60.29 ± 2.75 s^−1^ mΜ^−1^) (Table 1), indicating a shift in substrate selectivity.

The Ser175 residue of Pq3‐O‐UGT2 specifically contributes to the recognition of F2, and the S175A mutation decreased catalytic activity toward F2 but not Rh2 (Figure 6B,C). This mutant was subsequently combined with other mutants to further enhance activity toward Rh2 while reducing activity toward F2 (Table S2, Supporting Information). Interestingly, most combinations resulted in decreased catalytic activity toward Rh2 compared to individual mutations alone. However, when S175A was combined with L181W, the resulting double mutant displayed an increase in catalytic efficiency toward Rh2 (126.40 ± 2.93 s^−1^ mΜ^−1^) relative to L181W alone, while catalytic efficiency toward F2 (54.29 ± 2.05 s^−1^ mΜ^−1^) further decreased. This resulted in a catalytic efficiency toward Rh2 ≈2‐fold greater than that toward F2. Both Ser175 and Leu181 are located at the N‐terminal end of the Nα6 helix, near the top of the acceptor binding pocket (Figure 6B). This positioning suggests that the N‐terminal end of the Nα6 helix is sensitive to different sugar acceptors, consistent with the conformational changes observed in Nα6 upon superimposition of apo Pq3‐O‐UGT2 and its complexes with Rh2 and F2 (Figure S13A, Supporting Information).

At the bottom rear of the acceptor binding pocket, residues ILe146 and Leu362 were found to influence catalytic efficiency. Mutation of theses residues to leucine and alanine, respectively, increased catalytic activity toward Rh2 (Figure 5D; Table S2, Supporting Information). Similarly, the T139S and S143T mutations, located at the pocket's bottom, enhanced catalytic efficiency toward Rh2 by 2.2‐ and 2.5‐fold, respectively. While the ILe146/Leu362 double mutant maintained similar catalytic efficiency to the L362A single mutant, the T139S/S143T double mutant further enhanced catalytic efficiency compared to either single mutant alone. However, combining these double mutants with the S175A/L181W double mutant significantly reduced catalytic efficiency toward Rh2 (Table S2, Supporting Information).

The catalytic efficiencies of single, double, and quadruple Pq3‐O‐UGT2 mutations suggest the presence of synergistic effects in substrate recognition or catalysis among these residues. They contribute to a coordinated common function rather than acting independently. As a result, mutating multiple residues simultaneously can yield various outcomes: amplification of individual effects (e.g., S175A and L181W, T139S, and S143T), no additional changes (e.g., ILe146 and Leu362), or compromise of beneficial effects (e.g., quadruple mutants). Interestingly, synergistic enhancement is more likely when mutations occur in close proximity, such as Ser175 and Leu181 at the N‐terminal end of the Nα6 helix, or T139S and S143T at the bottom of the acceptor binding pocket. In contrast, systematic compromise tends to occur when mutations involve residues located in different regions, as observed with quadruple mutants. Whether these observations specific to Pq3‐O‐UGT2 are applicable to other systems remains to be investigated further.

### 2.8 The Acceptor Promiscuity of Pq3‐O‐UGT2

**Figure 7:**
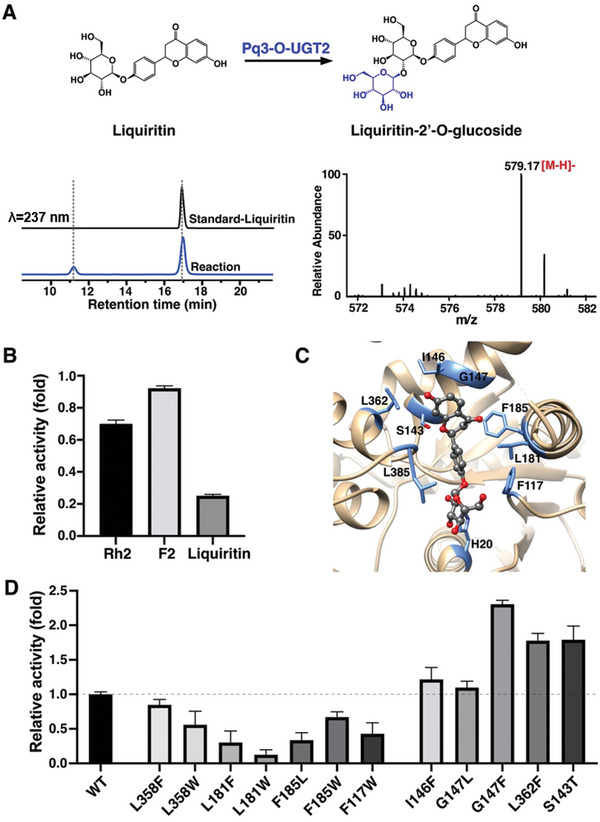
Pq3‐O‐UGT2 catalyzes the glycosylation of liquiritin. A) HPLC results of the reaction mixtures of liquiritin glycosylation by Pq3‐O‐UGT2 and mass spectra of the product. B) Pq3‐O‐UGT2 showed lower activity toward liquiritin than toward Rh2 and F2. Error bars represent standard deviation from 3 repeats. Lq, Liquiritin. C) Predicted binding mode of liquiritin to Pq3‐O‐UGT2 by docking. The key residues are shown as dark blue sticks, and liquiritin is shown as a stick‐and‐ball model, with the oxygen atoms colored in red. D) Relative activities of Pq3‐O‐UGT2 mutants toward liquiritin. Error bars represent standard deviation from 3 repeats.

To explore the substrate specificity of Pq3‐O‐UGT2s, we examined its catalytic activity toward a small library of mono‐glucosides and di‐glucosides of ginsenosides and flavonoids (Figures S17 and S18, Supporting Information). Interestingly, Pq3‐O‐UGT2 exhibited no activity toward other ginsenosides or notoginsenosides that share the common ginsenoside aglycone, indicating that the presence of C3‐O‐glucose, rather than other mono‐glucose groups, is essential for substrate recognition. In addition, the additional sugar rings present at the tail of ginsenosides (relative to F2) may induce spatial conflicts with the rear of the acceptor binding pocket. Surprisingly, Pq3‐O‐UGT2 catalyzed the β(1‐2) glycosylation of the C3‐O‐glucose of liquiritin (Lq) to generate liquiritin‐2′‐O‐glucoside (**Figure** **7**A; Figure S19, Supporting Information), albeit with much lower catalytic activity toward Lq compared to Rh2 and F2 (Figure 7B), demonstrating that Pq3‐O‐UGT2 has a broader acceptor spectrum that extends beyond ginsenosides.

Liquiritin is considerably smaller than Rh2 and features two oxygen atoms in its aglycone. To investigate its recognition mechanism by Pq3‐O‐UGT2, we docked Lq into the acceptor binding pocket (Figure 7C) and examined the catalytic activity of various Pq3‐O‐UGT2 mutants toward Lq (Figure 7D). Interestingly, mutations that increased catalytic activity toward Rh2 by “space‐filling” the binding pocket (Figure 5C) generally decreased catalytic activity toward Lq. This could be attributed to the oxygen atoms in Lq's aglycone, whose hydrophilicity complicates interactions with the hydrophobic residues in the central part of the acceptor binding pocket.

At the rear of the acceptor binding pocket, however, mutations introducing larger side chains enhanced the catalytic activity of Pq3‐O‐UGT2 toward Lq. Notably, a mutation at Gly147, located in the middle of Nα5, to phenylalanine increased catalytic activity by more than 2‐fold. These results suggest that hydrophobic interactions at the rear of the Pq3‐O‐UGT2 acceptor binding pocket play a significant role in the recognition of Lq. In contrast, the highly hydrophobic environment in the central region of the binding pocket disfavors acceptors with polar groups in their aglycone.

## 3 Discussion

Ginsenosides are a group of tetracyclic triterpenoids with significant bioactivity. Understanding the mechanisms underlying their recognition and glycosylation by UGTs is crucial for elucidating the structure‐function relationship of ginsenoside UGTs and enhancing their catalytic performance. Here, we solved the crystal structures of ginsenoside glycosyltransferase Pq3‐O‐UGT2 in its apo form and in complex with Ginsenoside Rh2 and F2. These structures reveal that Pq3‐O‐UGT2 binds its acceptors through an Nα5‐orientated binding pocket (Figure 3), similar to UGT91C1. In contrast, other UGTs that catalyze large substrates typically utilize an Nα3‐orientated pocket for acceptor binding.

This difference in acceptor binding pocket orientation is closely related to the conformation of the Nα5‐Nα6 linker (Figure 4). A hairpin structure in the Nα5‐Nα6 linker appears to prevent the formation of an Nα5‐orientated binding pocket, whereas the absence of this hairpin facilitates its formation. Notably, the recently reported SgUGT94‐289‐3, which also lacks the Nα5‐Nα6 hairpin, features an Nα5‐orientated acceptor binding pocket,^[^ ^47^ ^]^ further supporting this hypothesis.

Given the remarkable accuracy of AlphaFold2 in structure prediction, we investigated whether AlphaFold2‐predicted structures, in combination with analyses of the Nα5‐Nα6 linker conformation, could serve as a predictive tool for identifying acceptor binding pocket orientation. Interestingly, the Nα5‐Nα6 linker in the AlphaFold2‐predicted structure of Pq3‐O‐UGT2 aligns well with its crystal structure and similarly lacks the Nα5‐Nα6 hairpin (Figure S20A, Supporting Information). Moreover, the AlphaFold2‐predicted structure of UGT91C1 also lacks the Nα5‐Nα6 hairpin, consistent with its crystal structure. The low confidence score in this region of UGT91C1 (Figure S20B, Supporting Information) suggests its flexibility and inability to form a stable hairpin structure. Conversely, the Nα5‐Nα6 hairpin is present in all AlphaFold2‐predicted structures of UGT76G1, UGT73P12, UGT74AN2, and UGT74AN3 (Figure S16A, Supporting Information). The predicted models of these hairpin‐containing structures align well with their respective crystal structures, with high confidence (Figure S16B, Supporting Information), indicating the reliability of using AlphaFold2‐predicted models to analyze the Nα5‐Nα6 linker conformation. Therefore, analyzing AlphaFold2‐predicted structures, particularly the Nα5‐Nα6 linker conformation, could provide a reliable method for predicting the orientation of acceptor binding pocket in UGTs. However, additional studies involving acceptor‐bound complex structures and targeted mutagenesis are required to validate and refine this approach.

The Nα3‐ and Nα5‐orientated binding pockets discussed in this paper pertain specifically to large acceptors, such as terpenoids and sterols. These molecules are characterized by rigid aglycone backbones and elongated shapes, necessitating spacious and extended acceptor binding pockets for proper accommodation. In contrast, small acceptors like simple phenols require a compact space around the catalytic center, typically involving only a few residues in their recognition.^[^ ^25^, ^26^, ^27^, ^53^, ^54^ ^]^ Flavonoids and other polyphenols, despite their extended structures, are significantly smaller than terpenoids and sterols. Due to their multiple hydroxy groups and potential glycosylation sites, including hydroxy groups and carbon atoms, the orientations of these molecules in the acceptor binding pocket are highly variable (Figure S21, Supporting Information). Additionally, these acceptor molecules are often deeply buried within the acceptor binding pocket, with the Nα5‐Nα6 linker in these UGTs forming a hairpin structure that acts as the top lid of the pocket. An exception is UGT89C1, which has a short linker (6 residues) connecting the Nα5 and Nα6 helices. This short linker results in a wide opening on the top surface (Figure S21, Supporting Information). In contrast to Pq3‐O‐UGT2, the short Nα5‐Nα6 linker in UGT89C1 leads to the formation of a small and shallow acceptor binding pocket.

Interestingly, capsaicin, a polyphenol amide with a long, extended, and hydrophobic tail, resembles terpenoids in its structural features. However, its backbone is thinner and more flexible. The complex structure of PaGT3 with capsaicin (PDB ID: 7VEK) reveals a binding pocket for capsaicin that is similar to the one observed for Rh2 in Pq3‐O‐UGT2, despite PaGT3 featuring an Nα5‐Nα6 hairpin (Figure S21, Supporting Information). However, in this complex structure, the electron density of capsaicin is notably weak and present in only one of the two protomers in the asymmetric unit.^[^ ^39^ ^]^ Furthermore, the majority of capsaicin lies beneath a small helix located between the Nα5‐Nα6 hairpin and the N‐terminus of Nα6 (Figure S21, Supporting Information). This conformation raises questions about how capsaicin enters the pocket, given the constrained nature of this region. Without mutagenesis analysis of the acceptor‐interacting residues, further validation of the capsaicin binding pocket in PaGT3 is necessary to confirm its structural and functional relevance.

Hydrophobic interactions play a pivotal role in Rh2 recognition by Pq3‐O‐UGT2 (Figure 5). In contrast, only a single hydrogen bond is identified between the glucose moiety of Rh2 and Asn118, which is non‐essential for Rh2 recognition (Figure 5B). Similarly, the recognition of digitoxigenin by UGT74AN2 and the binding of cardiotonic steroids to UGT74AN3 are primarily driven by hydrophobic interactions.^[^ ^24^, ^45^ ^]^ However, for UGTs that catalyze simple phenols and polyphenols, both hydrophobic interactions and hydrogen bonds are crucial for acceptor recognition due to the presence of hydrophobic rings and multiple hydroxy groups in the acceptors.^[^ ^30^, ^32^, ^37^, ^40^, ^55^ ^]^

Hydrophobic recognition of acceptors often leads to poor regiospecificity, as observed in UGT76G1^[^ ^42^ ^]^ and UGT91C1.^[^ ^41^ ^]^ Both UGT76G1 and UGT91C1 catalyze glycan chain extension on different ends of the steviol aglycone. While hydrogen bonds play a role in stabilizing the glucose moiety of the steviol glucoside acceptors.^[^ ^45^, ^46^ ^]^ the hydrophobic recognition of the steviol aglycone is primarily responsible for poor regiospecificity. Additionally, the pseudo‐two‐fold symmetry of the steviol aglycone and the loose‐fitting hydrophobic pocket facilitates the binding of the steviol aglycone in two orientations.^[^ ^41^ ^]^ In contrast, Pq3‐O‐UGT2 demonstrates high regiospecificity toward its tested acceptors. One factor contributing to this specificity is its ability to catalyze glycan chain extension on the monoglucose of Rh2, where the position of the monoglucose on the aglycone determines its orientation in the catalytic center. Moreover, Rh2 snugly fits into the inner space of the acceptor binding pocket, with mutations of Phe117 and Phe185 imposing a strict “space‐filling” effect on catalytic activity, and mutations of Gly147 and Leu362 to residues with larger side chains reducing catalytic activity (Figure 5C,D). The limited inner space of the acceptor binding pocket for Rh2 also ensures the exclusive generation of a single sole product, Rg3. For F2, which features 2 monoglucoses on its aglycone, both hydrophobic interactions and hydrogen bonds play critical roles in ensuring high regiospecificity (Figure 6). The dual contributions of these interactions provide a mechanistic basis for the regiospecific recognition of F2 by Pq3‐O‐UGT2.

Screening a small library revealed that Pq3‐O‐UGT2 catalyzes the glycosylation of liquiritin, a flavonoid mono‐glycoside with various health benefits.^[^ ^56^ ^]^ Molecular docking and enzyme activity analysis highlight the critical role of residues located at the rear of the acceptor binding pocket in liquiritin recognition (**Figure** **7**). Although Pq3‐O‐UGT2 exhibited lower catalytic activity toward liquiritin compared to Rh2 and F2, this discovery expands its substrate range from ginsenosides to flavonoid glycosides. Further exploration of additional compounds is expected to broaden the substrate spectrum of Pq3‐O‐UGT2.

Guided by the complex structures of Pq3‐O‐UGT2‐Rh2 and Pq3‐O‐UGT2‐F2, several mutants with more than a 2‐fold increase in catalytic efficiency were successfully generated through mutagenesis of residues lining the acceptor binding pocket. Among these, the S175A/L181W mutant demonstrated significantly enhanced catalytic activity toward Rh2 and reduced activity toward F2, with the catalytic efficiency for Rh2 being twice that of F2. This engineered Pq3‐O‐UGT2 enzyme holds great potential for improving the production of the rare ginsenoside Rg3. Further optimization, such as enhancing its thermostability,^[^ ^57^, ^58^ ^]^ could further increase its catalytic performance and industrial applicability. Additionally, this engineered enzyme could be utilized in vitro enzymatic systems, where advanced immobilization techniques, such as Metal‐Organic Framework (MOF) systems,^[^ ^59^, ^60^, ^61^ ^]^ could improve both its catalytic efficiency and reusability. Given the demonstrated high‐yield production of Rh2,^[^ ^18^ ^]^ an in vitro enzymatic approach using the engineered Pq3‐O‐UGT2 enzyme represents a more promising and efficient method for Rg3 production compared to cell factory‐based strategies.

## 4 Experimental Section

### Chemicals and Reagents

20(S)‐Ginsenoside Rh2, Ginsenoside F2, liquiritin, Ginsenoside F1, Ginsenoside Rg1, Ginsenoside Rg2, Ginsenoside Rg3, Ginsenoside Mb, Notoginsenoside R1, Notoginsenoside R2, baicalin, vitexin, puerarin, cyanidin 3‐glucoside, and Ginsenoside Rd were obtained from PUSH BIO‐TECHNOLOGY (Chengdu, Sichuan, China). UDP‐glucose (UDP‐Glc) was purchased from RHAWN (Shanghai). Crystallization screening kits were purchased from Hampton Research (Laguna Niguel, CA). All other reagents were acquired from Sigma–Aldrich.

### Expression and Purification of Pq3‐O‐UGT2

The expression and purification of Pq3‐O‐UGT2 (GenBank: ALE15280.1) were conducted following a previously published paper.^[^ ^49^ ^]^ Briefly, Pq3‐O‐UGT2 was cloned into the pET‐28a vector with a C‐terminal 6× His tag and a TEV cleavage site and expressed using the *E. coli* Rosetta (DE3) strain. Cells were harvested, resuspended in lysis buffer (50 mM Tris‐HCl, pH 8.0, 150 mM NaCl, 1 mM PMSF, 0.5% Triton X100), and subjected to sonication for lysis. Subsequently, cell debris was removed by centrifugation at 26000 g and 4 °C for 30 min. The resulting supernatant was loaded onto pre‐equilibrated Ni‐NTA affinity resin (GenScript, Nanjing, China), washed extensively with wash buffer (50 mM Tris‐HCl, pH 8.0, 150 mM NaCl, 30 mM imidazole), and eluted with elution buffer (50 mM Tris‐HCl, pH 8.0, 150 mM NaCl, 500 mM imidazole). The eluted sample underwent additional purification through a HiTrap Q FF column (GE Healthcare), a Mono Q^T^ 5/50 column (GE Healthcare), and a Superdex 200 Increase 10/300 column (GE Healthcare). All protein samples were validated by SDS‐PAGE. The purified Pq3‐O‐UGT2 was flash‐frozen using liquid nitrogen and stored at −80 °C for further use. The expression of Pq3‐O‐UGT2 mutants followed a similar protocol as the wild‐type protein.

### Crystallization

The crystallization of Pq3‐O‐UGT2 was performed as described in our previously published work.^[^ ^49^ ^]^ The apo structure of Pq3‐O‐UGT2 was obtained from crystals co‐crystallized with UDP‐Glc. However, the electron density for UDP‐Glc in the sugar donor binding pocket was not resolved. The complex structures of Pq3‐O‐UGT2 with Rh2 and F2 were derived from crystals co‐crystallized with Rh2 and F2, respectively.

### Crystal Structure Determination and Refinement

X‐ray diffraction data sets were collected at beamlines 18U1, 19U1, and BL10U2 at the Shanghai Synchrotron Radiation Facility. The diffraction data underwent integration and scaling using HKL‐2000.^[^ ^62^ ^]^ Molecular replacement was employed for the structure solution, utilizing PaGT2 (PDB ID: 6JEM) as a search model through Phaser^[^ ^63^ ^]^ from the CCP4 suite.^[^ ^64^ ^]^ Manual model building and subsequent refinement were carried out using Coot^[^ ^65^ ^]^ and Phenix.Refine.^[^ ^66^ ^]^ The restraint files of Rh2 and F2 were generated by Phenix.eLBOW.^[^ ^67^ ^]^ Model statistics were validated using MolProbity.^[^ ^68^ ^]^ The comprehensive data collection and refinement statistics are summarized in Table S1 (Supporting Information). All graphical representations were crafted using the UCSF Chimera^[^ ^69^ ^]^ program.

### Molecular Docking

The crystal structure of Pq3‐O‐UGT2 in complex with Rh2 (PDB ID: 8K08, chain B) served as the basis for molecular docking studies involving UDP‐Glc and liquiritin. Structure files for UDP‐Glc and liquiritin were retrieved from Pubchem. For liquiritin docking, Rh2 was omitted from the complex structure prior to the docking procedure. Molecular docking was conducted using AutoDock Vina^[^ ^70^ ^]^ within UCSF Chimera.^[^ ^69^ ^]^ In the case of UDP‐Glc docking, the grid center was determined through alignment with other UGT structures, and the search space was set at 20 × 20 × 20 Å. For liquiritin docking, the grid center was positioned based on the center of Rh2, and the search space was set at 20 × 20 × 20 Å. All other docking parameters were maintained at their default settings. Multiple models were generated by AutoDock Vina for both UDP‐Glc and liquiritin docking, with the most plausible models selected for subsequent analysis.

### Site‐Directed Mutagenesis

For the generation of Pq3‐O‐UGT2 mutants, overlap PCR was employed using mutation primers (refer to Table S3, Supporting Information) and the wild‐type pET28a‐Pq3‐O‐UGT2 plasmid as a template. The PCR assays were carried out utilizing Pfx DNA polymerase (Genscript, Nanjing, China). Subsequently, the amplified PCR products were ligated into NcoI/BamHI double‐digested pET28a vectors. The accuracy of all mutations was verified through sequencing.

### Conversion Rate and Relative Activity Analysis

To assess the relative activity of Pq3‐O‐UGT2 and its mutants toward Rh2 or F2, all proteins, including the wildtype and mutants, were prepared using a similar protocol and purified to the same level of purity. All the mutants were stable and folded properly, as evidenced by their similar size exclusion chromatography profile. The enzyme activity assay was conducted in a 100 µL system comprising 2 µg purified enzyme, 0.5 mM acceptor substrate, 5 mM UDP‐Glc, and 50 mM Tris‐HCl at pH 8.0. The reaction mixtures were incubated at 37 °C for 30 min and then quenched by adding 100 µL of methanol. The conversion rate was determined using HPLC and calculated by dividing the integrated peak area of the glycosylated product by the sum of the peak areas of the glycosylated product and the remaining substrate. All experiments were performed in triplicate. To determine the relative activity of the mutant enzymes compared to the wildtype, the conversion rates of the mutants were normalized to that of the wildtype enzyme.

For determining the conversion rate of Pq3‐O‐UGT2 with different substrates (Rh2, F2, and liquiritin), as well as evaluating the relative activity of Pq3‐O‐UGT2 wild type and mutants toward liquiritin, the enzyme activity assay was performed in a 100 µL system containing 10 µg purified enzyme, 0.5 mM individual acceptor substrate, 5 mM UDP‐Glc, and 50 mM Tris‐HCl at pH 8.0. The reaction mixtures were incubated at 37 °C for 12 h and then stopped by adding 100 µL of methanol. After termination, the reaction mixtures were centrifuged at 16200 g for 15 min. The supernatant was filtered through a 0.22‐µm microporous membrane and analyzed by high‐performance liquid chromatography (HPLC). All measurements were performed in triplicates.

### Library Screening

The Pq3‐O‐UGT2 activity assay was performed in a 100 µL reaction buffer comprising 0.5 mM substrate, 5 mM UDP‐Glc, 100 mM phosphate buffered saline (pH 7.5), and 10 µg of purified enzyme. The reaction mixture was incubated at 37 °C for 12 h, and the reaction was stopped by adding an equal volume of methanol. The resulting mixture was centrifuged at 16200 g to remove impurities and subsequently analyzed by either HPLC or HPLC‐MS. Each substrate was measured in triplicate for accuracy and consistency.

### HPLC Analyses of the Catalytic Products

High‐performance liquid chromatography (HPLC) analyses of the catalytic products were performed using two systems: an Agilent TC‐C18 column (4.6 × 250 mm, 5 µm, Agilent, USA) connected to an Agilent 1260 HPLC system, and a Kromasil 100–5 C18 column (4.6 × 250 mm, 5 µm, Kromasil, Sweden) connected to a SHIMADZU HPLC system. A 20 µL aliquot of each reaction product was injected and analyzed at a flow rate of 1 mL mi^−1^n. The elution conditions were as follows: 1) 20(S)‐Ginsenoside Rh2: solvent A: water, solvent B: acetonitrile, gradient: 0–30 min, 30% B‐90% B. 2) Ginsenoside F2: solvent A: water, solvent B: acetonitrile, gradient: 0–10 min, 30% B‐70% B; 10–14 min, 30% B‐70% B. 3) Liquiritin: solvent A: 0.04% formic acid solution, solvent B: methanol, gradient: 0–25 min, 15% B‐90% B. 4) Ginsenoside F1, Ginsenoside Rg1, Ginsenoside Rg2, Ginsenoside Rg3, Notoginsenoside R1, and Notoginsenoside R2: solvent A: water, solvent B: acetonitrile, gradient: 0–35 min, 20% B; 35–40 min, 20% B‐30% B; 40–50 min, 30% B‐31% B; 50–60 min, 31% B‐32% B; 60–70 min, 32% B‐45% B; 70–100 min, 45%B‐60% B; 100–110 min, 60% B; 110–111 min, 60% B‐20% B; 111–120 min, 20%B. 5) Ginsenoside Mb: solvent A: water, solvent B: acetonitrile, gradient: 0–14 min, 30% B‐100% B. 6) Baicalin: solvent A: water, solvent B: methanol, isocratic: 0–15 min, 35% B. 7) Vitexin: solvent A: water, solvent B: methanol, isocratic: 0–20 min, 20% B. 8) Puerarin: solvent A: water, solvent B: methanol, isocratic: 0–20 min, 25% B. 9) Cyanidin 3‐glucoside: solvent A: water, solvent B: methanol, gradient: 0–20 min, 12% B‐100% B.

### Determination of Kinetic Parameters

For the determination of kinetic parameters of Pq3‐O‐UGT2 with various substrates, 100 µL reactions were incubated at 37 °C for 15 min. The reaction mixtures comprised 100 ng of purified enzyme (Pq3‐O‐UGT2 or the mutants), 3 mM UDP‐Glc, and varying concentrations (20–600 µM) of sugar acceptor (Rh2 or F2). The reactions were terminated by adding an equal volume of methanol, followed by centrifugation at 16200 g for 15 min. The resulting supernatants were subjected to HPLC analysis. All experiments were performed in triplicate. The enzyme kinetic parameters were determined by quantifying the formation of glycosylated products, and the values of *K*_M_ and *k*_cat_/*K*_M_ were calculated using the Michaelis−Menten plot.

### LC‐MS Analysis of the Catalytic Products

A 100 µL reaction mixture containing 50 mM Tris‐HCl, pH 7.5, 5 mM UDP‐Glc, 0.5 mM substrate (liquiritin, F2, or Rh2), and 10 µg purified Pq3‐O‐UGT2 was incubated at 37 °C for 12 h in a glovebox to facilitate the glycosylation of substrates. Following incubation, the reaction was quenched with methanol and subjected to a 45 s denaturation in a boiling water bath to completely denature the protein. The precipitated protein was then removed by centrifugation at 16200 g for 10 min, and the resulting supernatant was filtered using a 0.22‐µm PES membrane. Subsequently, a 20 µL aliquot of the supernatant was subjected to LC‐MS analysis.

For the analysis of the glycosylation product of liquiritin, the supernatant was subjected to an Agilent 6420 Triple Quadrupole LC/MS instrument (Agilent Technologies) equipped with a C18 column (4.6 × 250 mm, 5 µm, Agilent, USA). The solvent system consisted of water (A) and acetonitrile (B), with the sample eluted through a linear gradient of 10%–90% B over 30 min, at a flow rate of 1 mL mi^−1^n. The products were detected by UV absorption at 237 nm, and product identities were confirmed by comparison with commercial standards and validated through positive mode electrospray ionization mass spectrometry.

For the analysis of the glycosylation product of F2, the supernatant was subjected to a Q Exactiye HF orbitrap mass spectrometer (ThermoFisher Scientific, USA) equipped with a C18 column (4.6 × 250 mm, 5 µm, Agilent, USA). The solvent system consisted of water (A) and acetonitrile (B), with the sample eluted through a linear gradient of 10%–90% B over 30 min, at a flow rate of 0.5 mL mi^−1^n. The products were detected by UV absorption at 203 nm, and product identities were confirmed by comparison with commercial standards and validated through positive and negative mode electrospray ionization mass spectrometry.

### NMR Spectroscopy of the Liquiritin Glycosylation Product

For the structural analysis of the glycosylation product of liquiritin by Pq3‐O‐UGT2, a scale‐up catalytic reaction (500 mL) was prepared. The reaction was terminated by adding an equal volume of methanol, followed by evaporation to 5–10 mL. Subsequently, the resulting solution was purified using a preparative HPLC system coupled with a reverse‐phase C18 column. After evaporation, the purified products were dissolved in 0.6 mL of MeOH‐*d*4 and transferred to an NMR tube (5.0 mm o.d. × 25 cm). Then, ^1^H‐NMR, ^13^C‐NMR, heteronuclear singular quantum correlation spectroscopy (HSQC), heteronuclear multiple‐bond correlation spectroscopy (HMBC), and heteronuclear multiple‐bond correlation spectroscopy (COSY) were performed using a 600 MHz NMR spectrometer.

### Data Analysis

Data analysis was conducted using Microsoft Excel 2019, and a graphical presentation was generated using GraphPad Prism 8. The results are expressed as the mean ± standard deviation (S.D.).

### Data Availability

The coordinates and structure factors have been deposited in the Protein Data Bank under accession codes 8JZQ, 8K08, and 8K09. All pertinent data are either presented in the paper, the Supporting Information file, or the source data file. The source data underpinning the kinetic parameters can be found in the Source Data file. Further data supporting the findings of this study are accessible from the corresponding author upon reasonable request.

## Conflict of Interest

The authors declare no conflict of interest.

## Author Contributions

Q.J., Y.L., and H.Z. contributed equally to this work and are co‐first authors. K.M., W.G., and J.W. performed conceptualization. Q.J., Y.L., H.Z., C.C., Y.G., Y.‐X.D., Y.‐Y.D, J.X., and X.J. performed investigation. Q.J., Y.L., H.Z., J.Z., and J.W. performed critical reagents. K.M., J.W., and W.G. performed project administration. K.M. and W.G. provide resources. Q.J. and K.M. Wrote the original draft. K.M., W.G., J.W., C.C., and B.L. Wrote, reviewed and edited the original draft. All authors contributed to the article and approved the submitted version.

## Supporting information



Supporting Information

## Data Availability

The data that support the findings of this study are available from the corresponding author upon reasonable request.
