# Supplementary material for: Structural Insights into the Substrate Recognition of Ginsenoside Glycosyltransferase Pq3‐O‐UGT2
Source: Adv Sci (Weinh). 2025 Jan 29;12(11):2413185. doi: 10.1002/advs.202413185 (PMC11923902; doi:10.1002/advs.202413185)
Supplement: Supplementary file 1 — Supporting Information [file ADVS-12-2413185-s001.docx]

Supplementary Materials for

**Structural Insights into the Substrate Recognition of Ginsenoside Glycosyltransferase Pq3-O-UGT2**

Qiushuang Ji *et al*.

*Corresponding author. Email: [kmei@tju.edu.cn](mailto:kmei@tju.edu.cn), [pharmgao@tju.edu.cn](mailto:pharmgao@tju.edu.cn), [drwangjuan@tju.edu.cn](mailto:drwangjuan@tju.edu.cn), [chengchen@tju.edu.cn](mailto:chengchen@tju.edu.cn)

**This PDF file includes:**

Figs. S1 to S21

Tables S1 to S3

**Supplement Results**

**
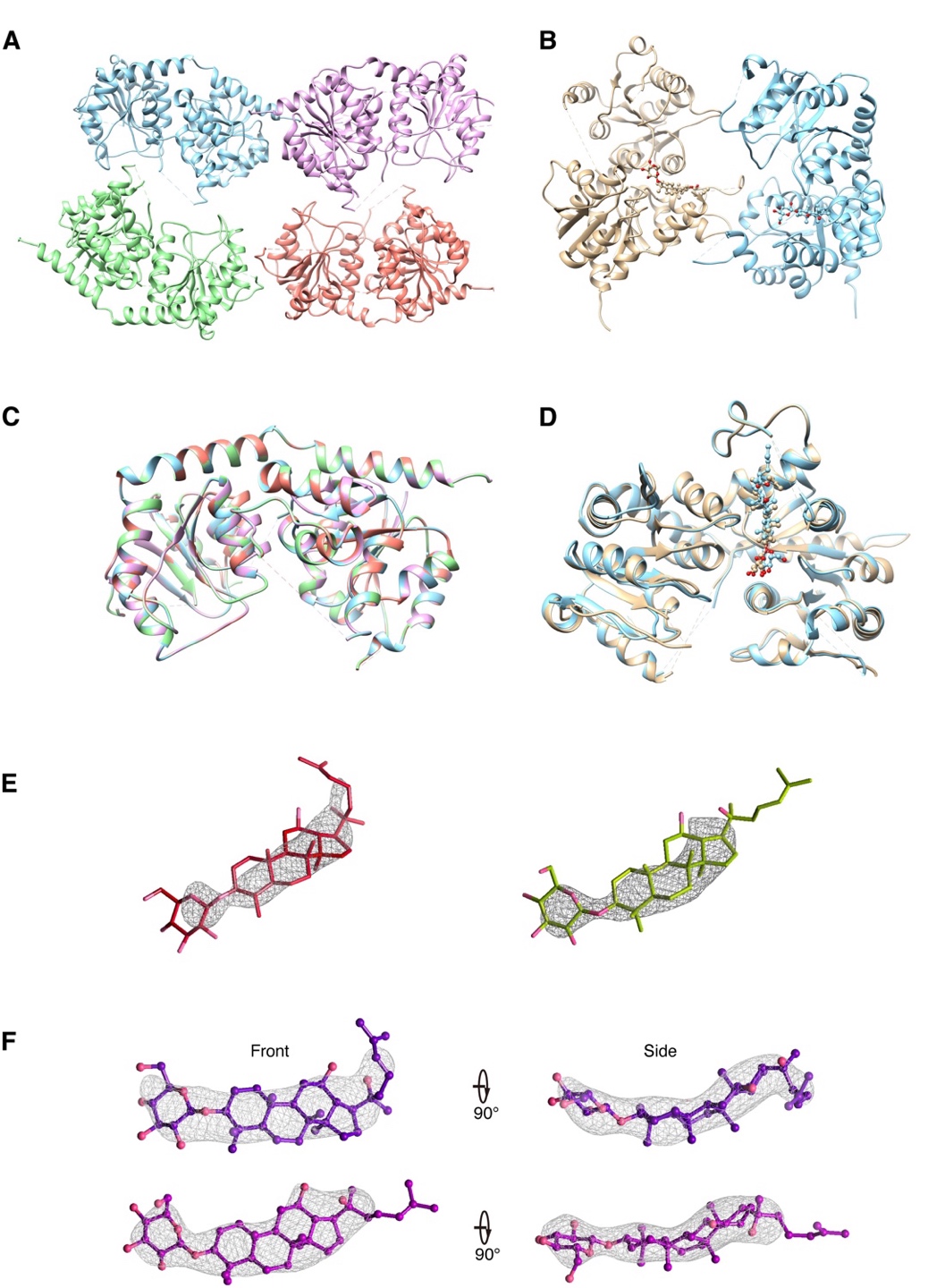
**

**Figure S1. Crystal structure of apo Pq3-O-UGT2 and its complex with Ginsenoside Rh2. A,** Four Pq3-O-UGT2 protomers in the asymmetric unit of the apo Pq3-O-UGT2 structure. **B,** Two Pq3-O-UGT2 protomers in the asymmetric unit of Pq3-O-UGT2 in complex with Rh2. Rh2 is depicted with stick-and-ball models. **C,** Alignment of the four Pq3-O-UGT2 protomers in apo Pq3-O-UGT2 structure. **D,** Alignment of the two Pq3-O-UGT2 protomers in the complex structure of Pq3-O-UGT2 and Rh2. Rh2 is depicted with stick models. **E,** The experimental Fo-Fc omit maps (contoured at 2.5σ) for Rh2 in the two protomers of the complex structure of Pq3-O-UGT2 and Rh2. **F**, The final refined 2Fo-Fc electron density maps (contoured at 1.5σ) for Rh2 in the two protomers of the complex structure.

**
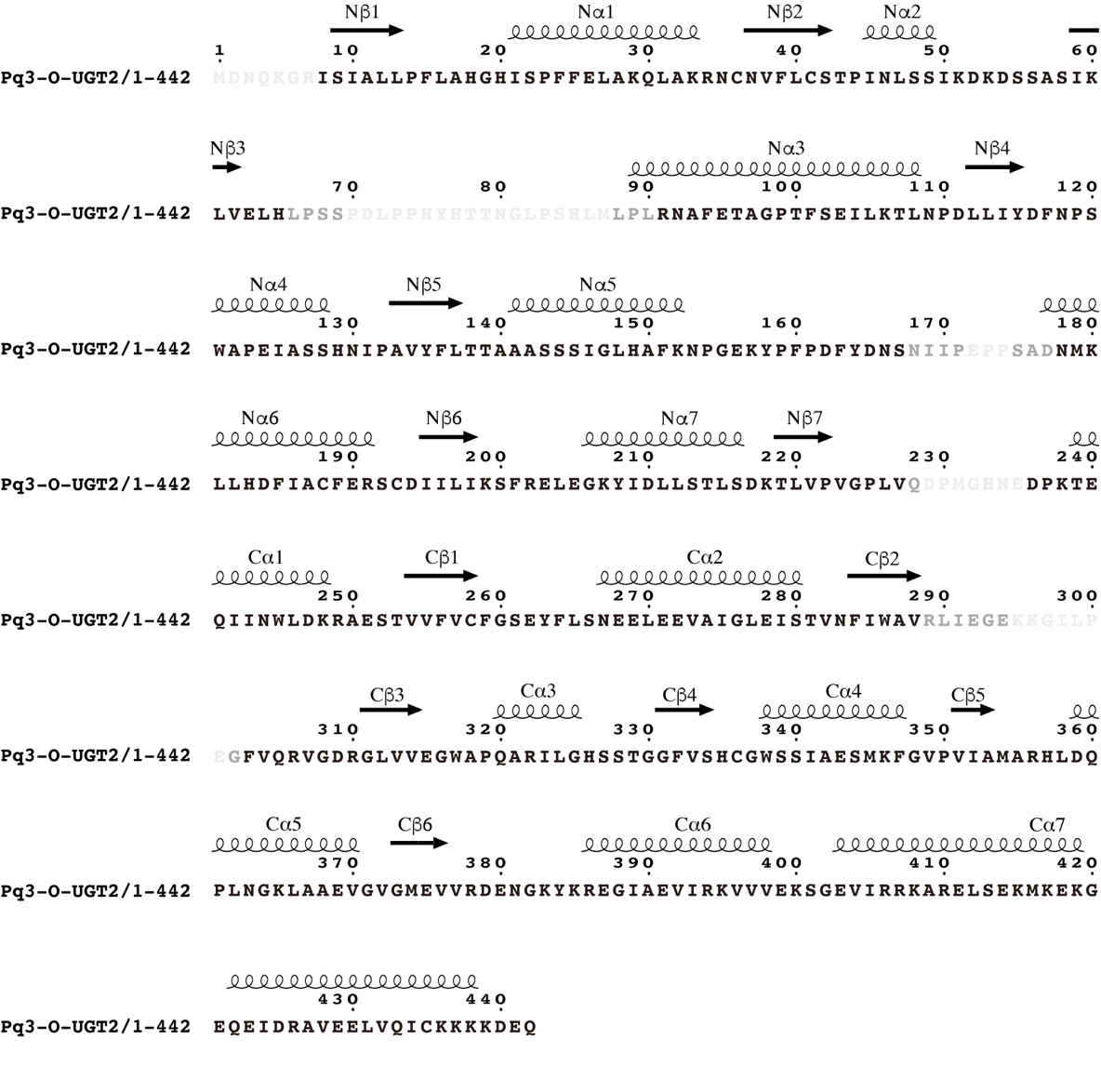
**

**Figure S2. Secondary structure of Pq3-O-UGT2.** The secondary structure of Pq3-O-UGT2 is annotated above the sequence. Unmodeled sequences in the apo form of Pq3-O-UGT2 are indicated in dark gray, while light gray denotes unmodeled sequences in both the apo form of Pq3-O-UGT2 and its complex with Rh2.


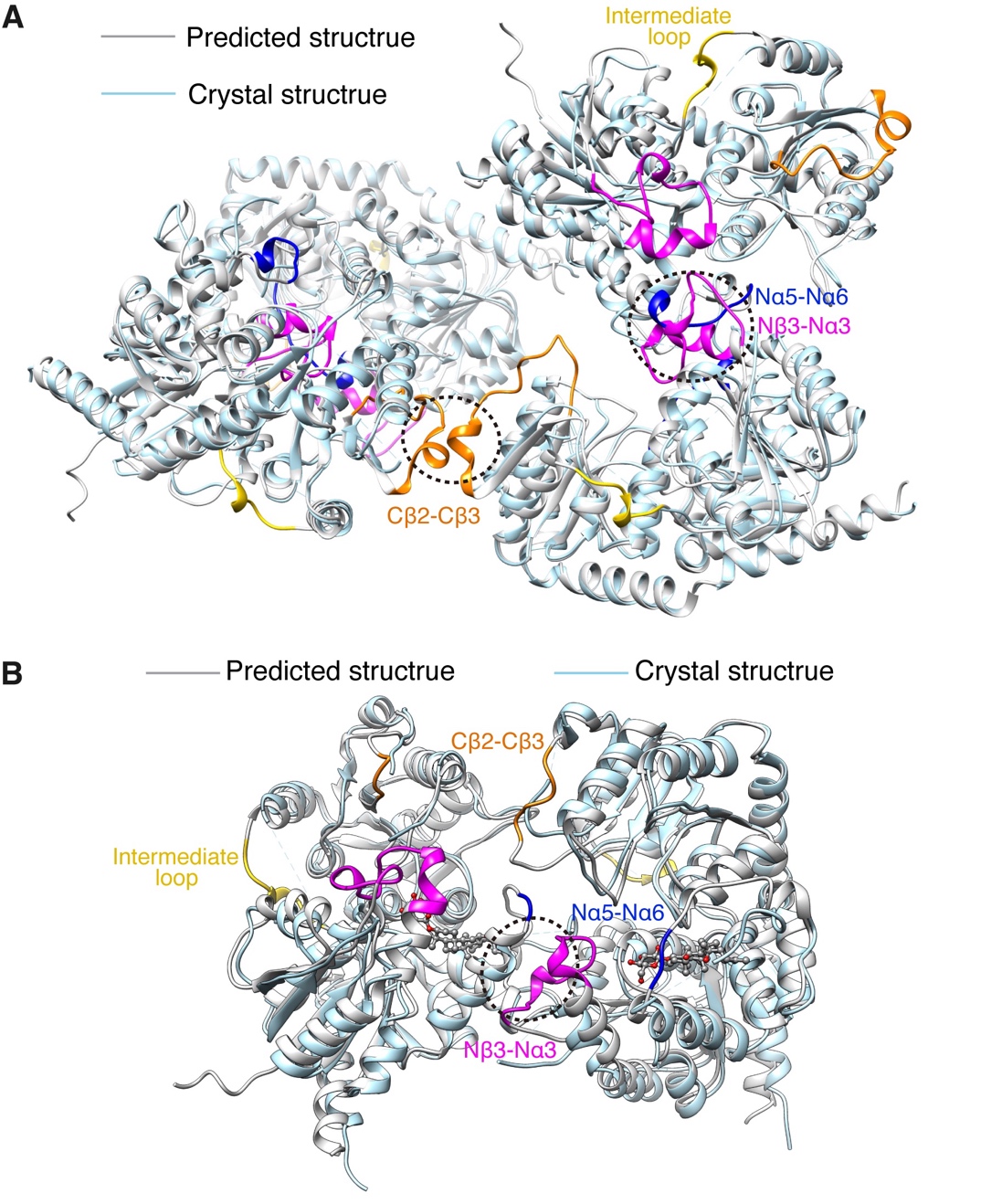


**Figure S3. Analysis of the missing density in the crystal structures of apo Pq3-O-UGT2 and its complex with Ginsenoside Rh2. A,** Alignment of the AlphaFold2-predicted Pq3-O-UGT2 structure to each of the protomers in the crystal structure of apo Pq3-O-UGT2. **B,** Alignment of AlphaFold2-predicted Pq3-O-UGT2 structure to each of the protomers in the crystal structure of Pq3-O-UGT2 in complex with Rh2. Segments of predicted structures, whose counterpart in the crystal structure is missing, are highlighted and labeled. Structures with potential spatial conflict are indicated by dotted circles.

**
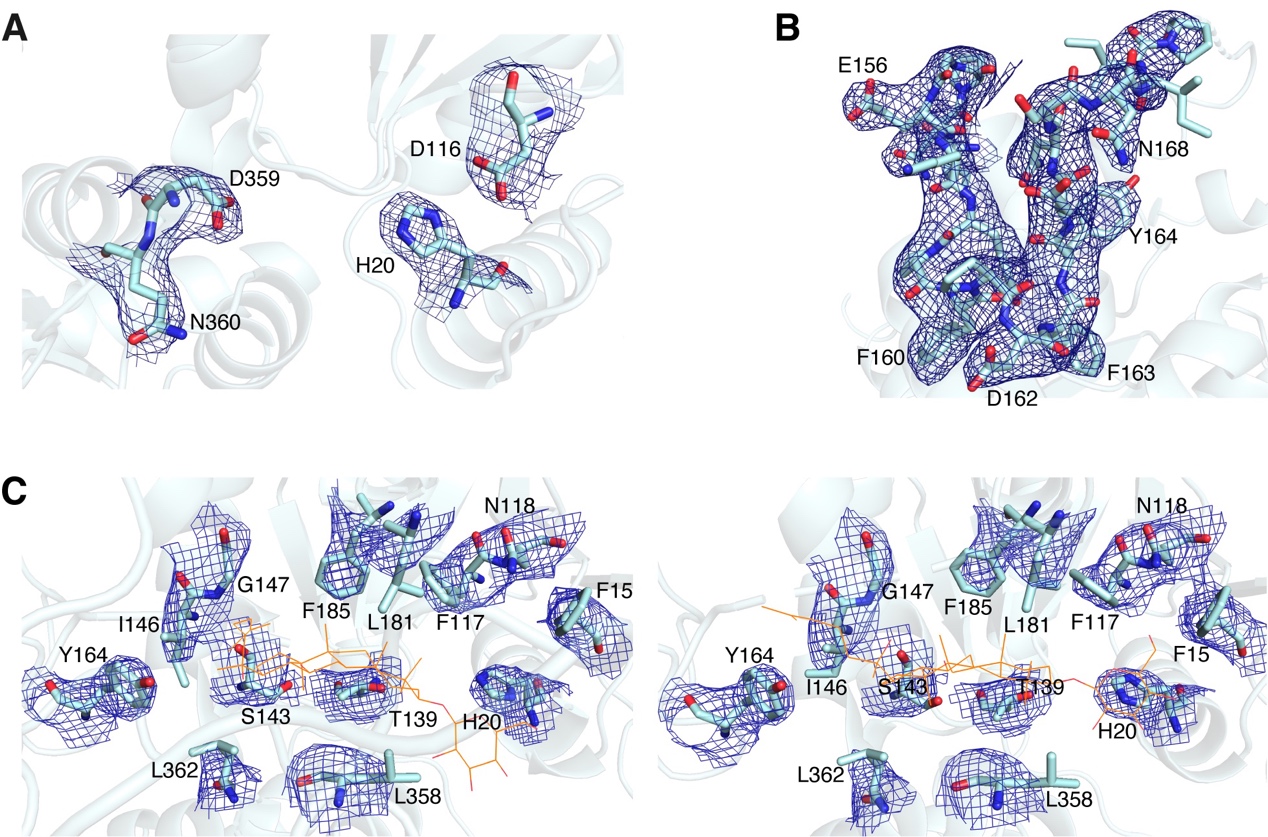
**

**Figure S4. Electron density maps of key residues and the Nα5-Nα6 linker of Pq3-O-UGT2.** **A,** 2Fo-Fc map of the residues involved in catalysis and donor sugar interaction. Related to **Figure 2**. **B,** 2Fo-Fc map of the Nα5-Nα6 linker. Related to **Figure 4**. **C**, 2Fo-Fc map of the residues in the acceptor binding pocket of Pq3-O-UGT2 in the two protomers of the complex structure. Rh2 is represented as an orange line model. Related to **Figure 5**. All maps are contoured at 1σ.

**
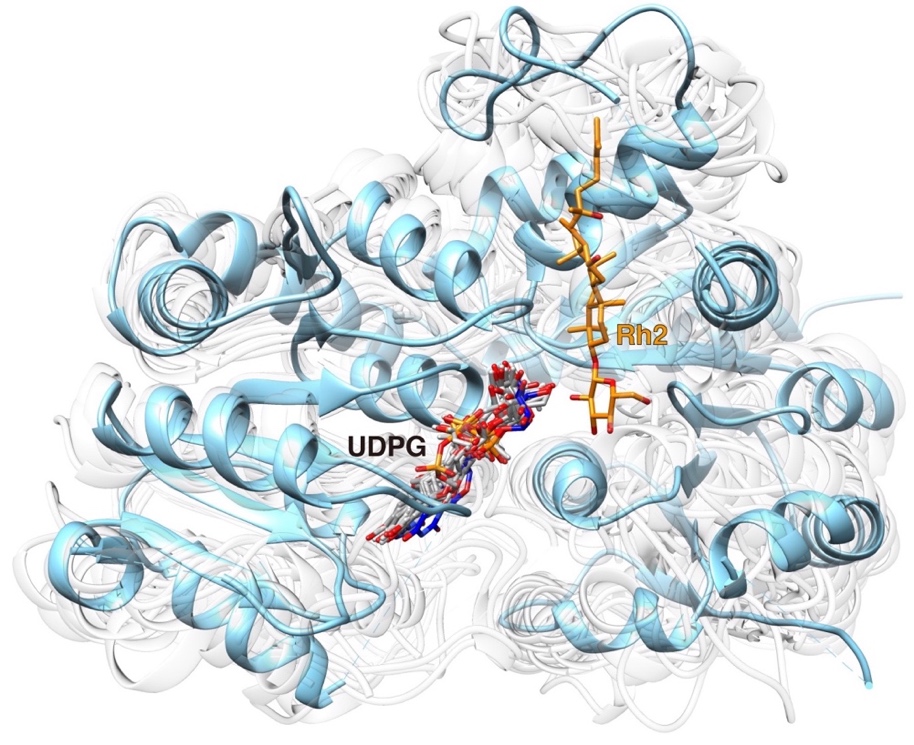
**

**Figure S5. Superimposition of Pq3-O-UGT2 with docked** **UDP-Glc onto crystal structures of other plant UGTs in complex with UDP-Glc**. Pq3-O-UGT2 is shown in light blue, and all other UGTs are shown in gray with semitransparency. All ligands are depicted as stick models. UDP-Glc in Pq3-O-UGT2 is colored in dark blue, and other UDP-Glc molecules are in dark gray, with oxygen atoms in red and phosphate atoms in orange. Rh2 is shown in orange with oxygen atoms in red. UDPG, UDP-Glc.

**
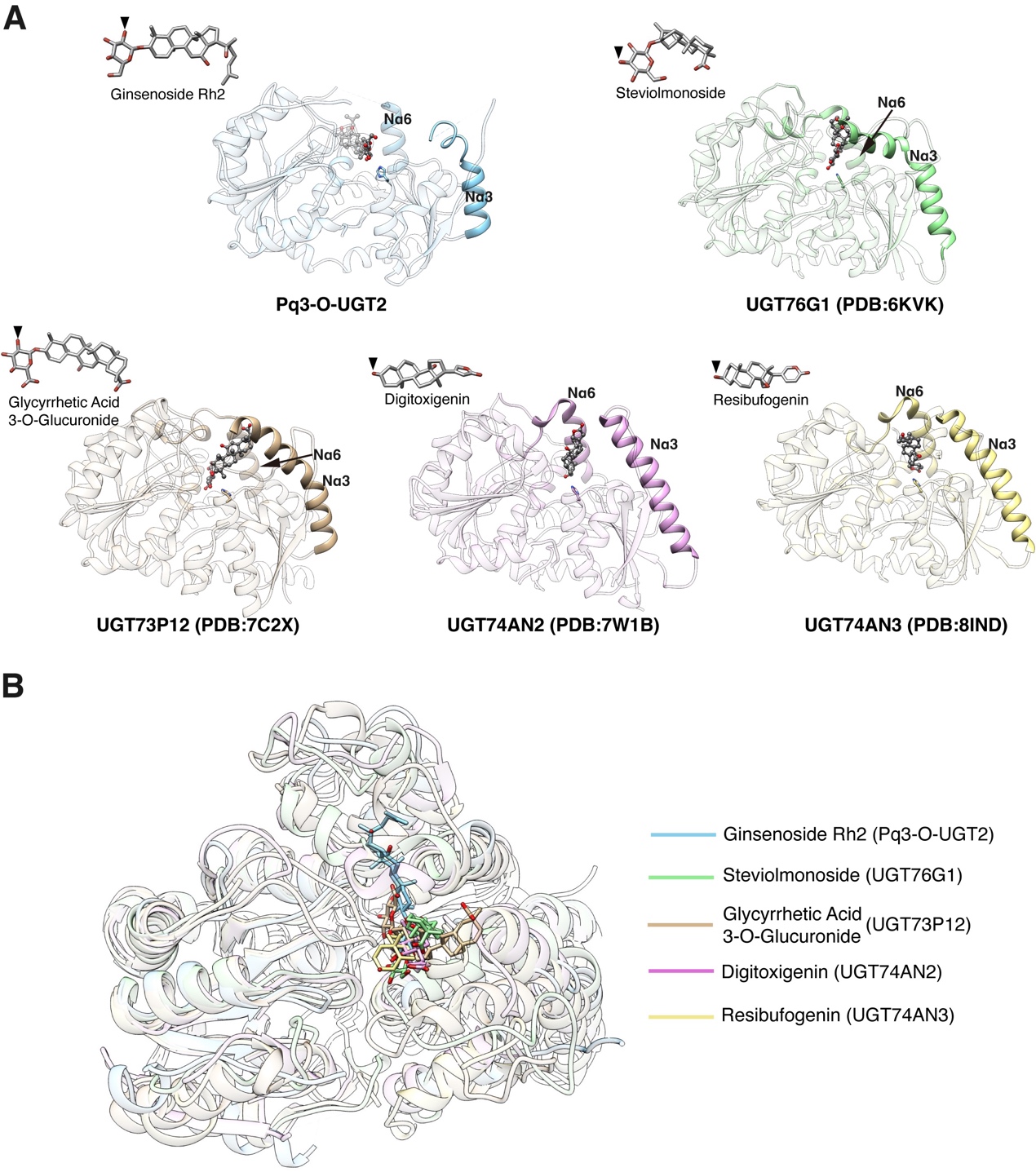
**

**Figure S6. Pq3-O-UGT2 binds to Rh2 with a distinctive orientation. A,** The binding of large and elongated acceptors to plant UGTs, shown in a perpendicular view to **Figure 3B**. **B,** Alignment of Pq3-O-UGT2, UGT76G1, UGT73P12, UGT74AN2, and UGT74AN3 with their representative acceptors. UGTs are shown as semitransparent ribbons. The acceptors are depicted as stick models as indicated.

**
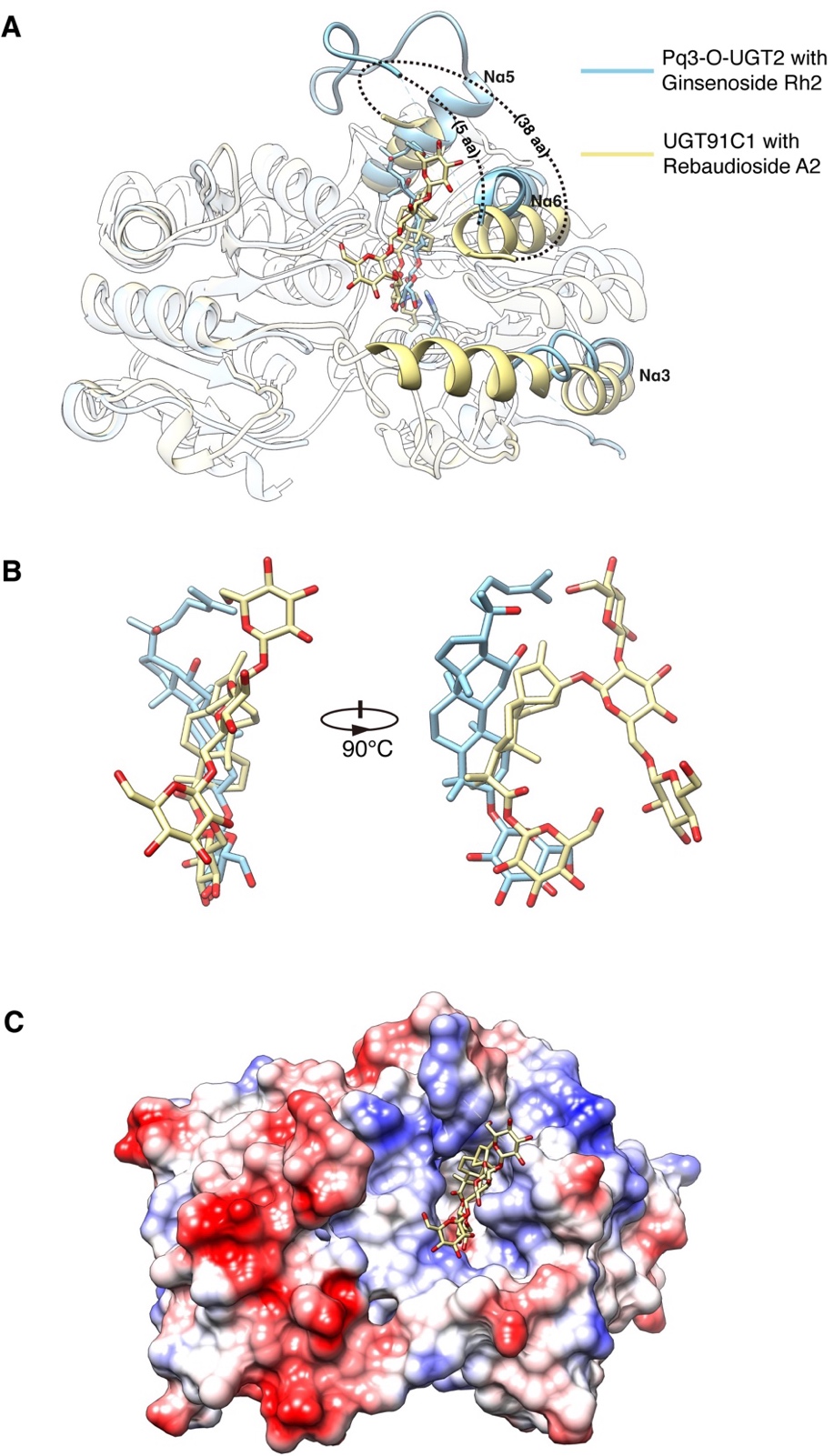
**

**Figure S7. Comparison of the acceptor binding pocket of Pq3-O-UGT2 and UGT91C1.** **A,** Alignment of Pq3-O-UGT2 and UGT91C1(PDB:7ES0) with bound acceptors. The acceptors are depicted as sticks. The Nα3, Nα5, Nα6 helices, and the Nα5-Nα6 linker are highlighted in the complex structures, with other parts presented in semitransparency. The missing structure for the Nα5-Nα6 linker is indicated. **B,** Zoom-in view of the acceptors in **A. C,** The binding pocket of UGT91C1. The surface is colored according to the electrostatic potential with the Coulombic Surface Coloring method. The acceptor is shown as a stick model.


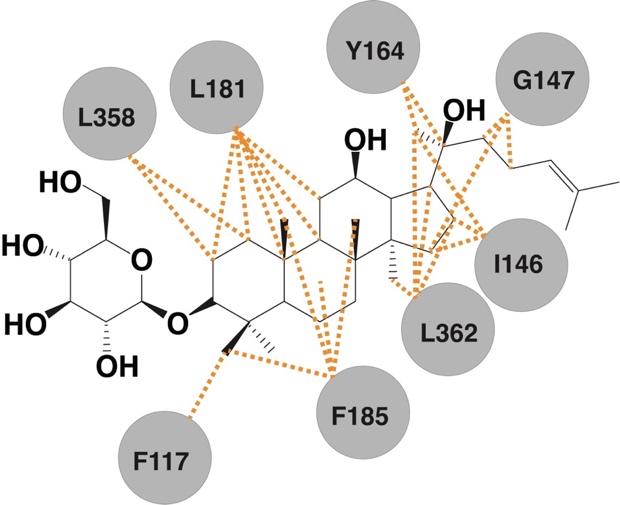


**Figure S8. Hydrophobic interaction analyses between Pq3-O-UGT2 and Rh2.** Dashed lines indicate distances within 5 Å between residues of Pq3-O-UGT2 and specific atoms of Rh2, highlighting key hydrophobic interactions.


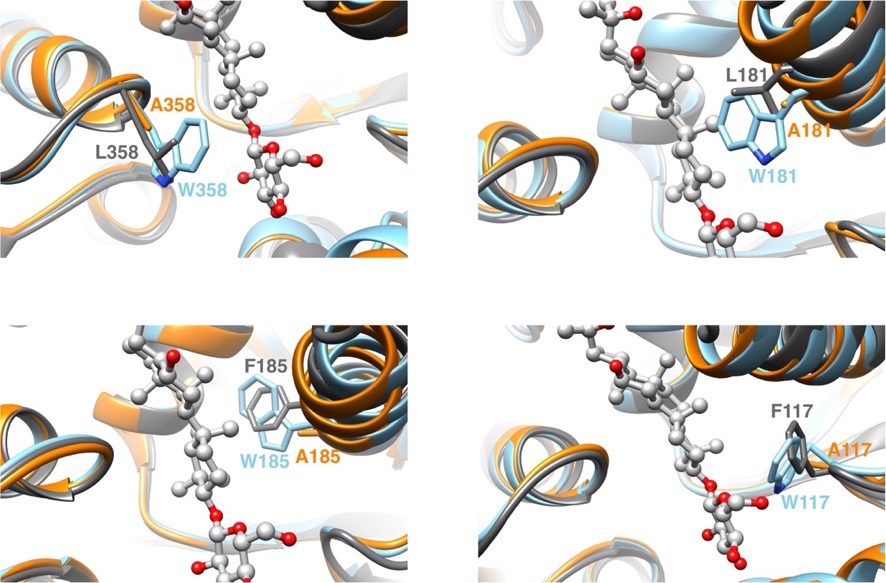


**Figure S9. AlphaFold2-predicted structures of Pq3-O-UGT2 with tryptophan and alanine single mutations**. The structures of Pq3-O-UGT2 with L358A, L358W, L181A, L181W, F185A, F185W, F117A, and F117W single mutations were predicted by AlphaFold2. The solved crystal structure of Pq3-O-UGT2 in complex with Rh2 is shown in dark gray, the alanine mutants are shown in orange, and the tryptophan mutants are shown in light blue. Rh2 is represented as a stick-and-ball model, and the target residues are shown as sticks.

**
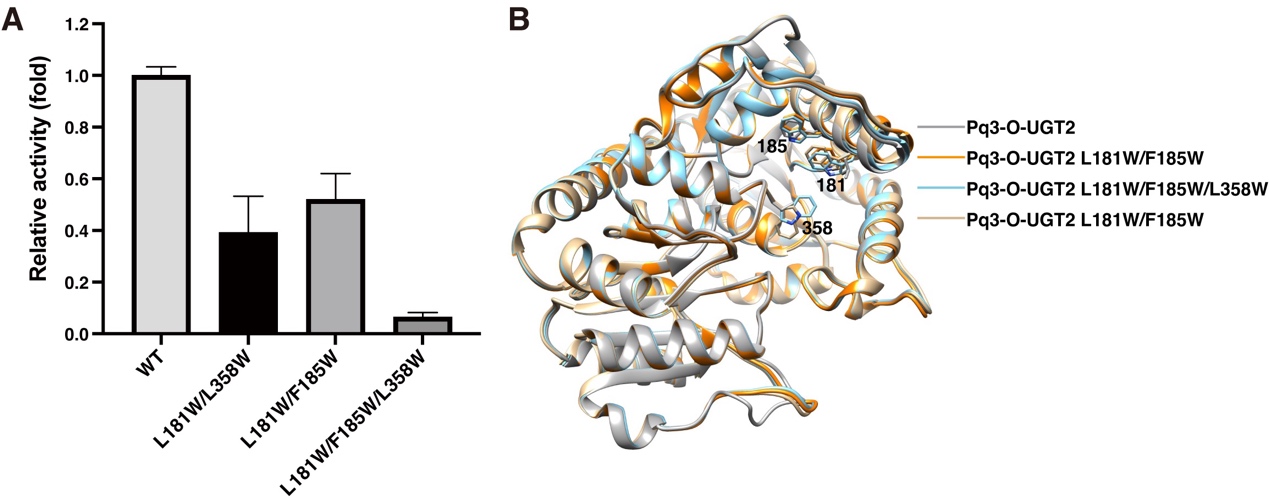
**

**Figure S10.** **Impact of space-filling mutations on the enzymatic activity of Pq3-O-UGT2. A,** Relative activities of Pq3-O-UGT2 mutants towards Rh2. Error bars represent the standard deviation from three repeats. **B,** AlphaFold2-predicted structures of Pq3-O-UGT2 with double or triple mutations. The 181^st^, 185^th^, and 358^th^ residues are highlighted and shown as stick representations.

**
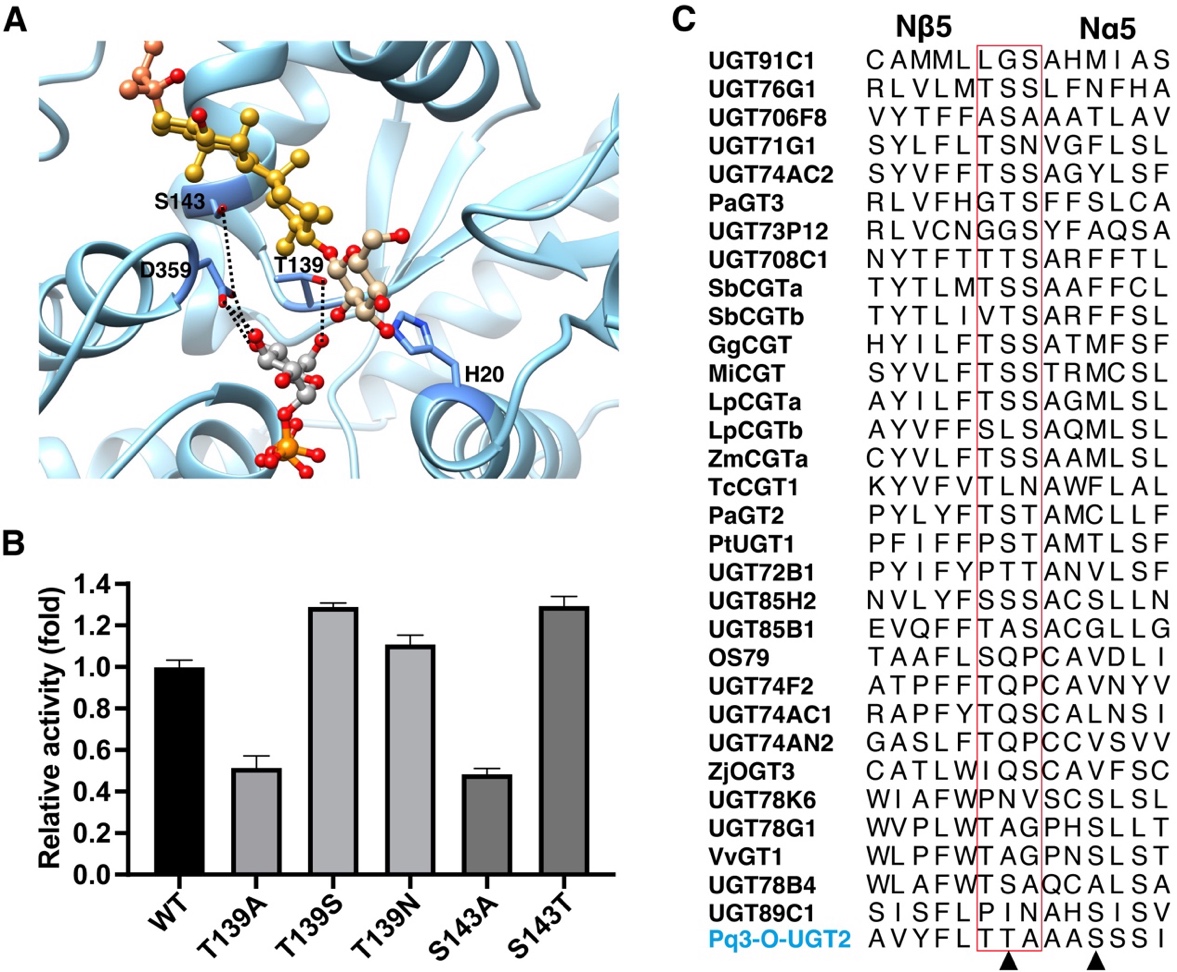
**

**Figure S11. Thr139 and Ser143 are involved in Pq3-O-UGT2 activity through interactions with the sugar donor. A,** Thr139 and Ser143 interact with UDP-Glc through hydrogen bonds. UDP-Glc was docked into the Pq3-O-UGT2 and Rh2 complex structure via AutoDock Vina. Hydrogen bonds are represented as dashed lines. UDP-Glc and Rh2 are represented as ball-and-stick models. The carbon, phosphate, and oxygen atoms of UDP-Glc are shown in gray, orange, and red, respectively. For Rh2, the glucose moiety is colored in tan, the tetracyclic ring in gold, the alky tail in coral, and oxygen atoms in red. **B,** Relative activities of Pq3-O-UGT2 mutants towards Rh2. Error bars represent the standard deviation from three repeats. **C,** Sequence alignment of plant UGTs with resolved structures, showing the Nβ5-Nα5 linker located at the bottom of the acceptor binding pocket. Sequence alignment was done with Jalview-Muscle. The Nβ5-Nα5 linker is indicated with a red box. Thr139 and Ser143 of Pq3-O-UGT2 are indicated with arrowheads.

**
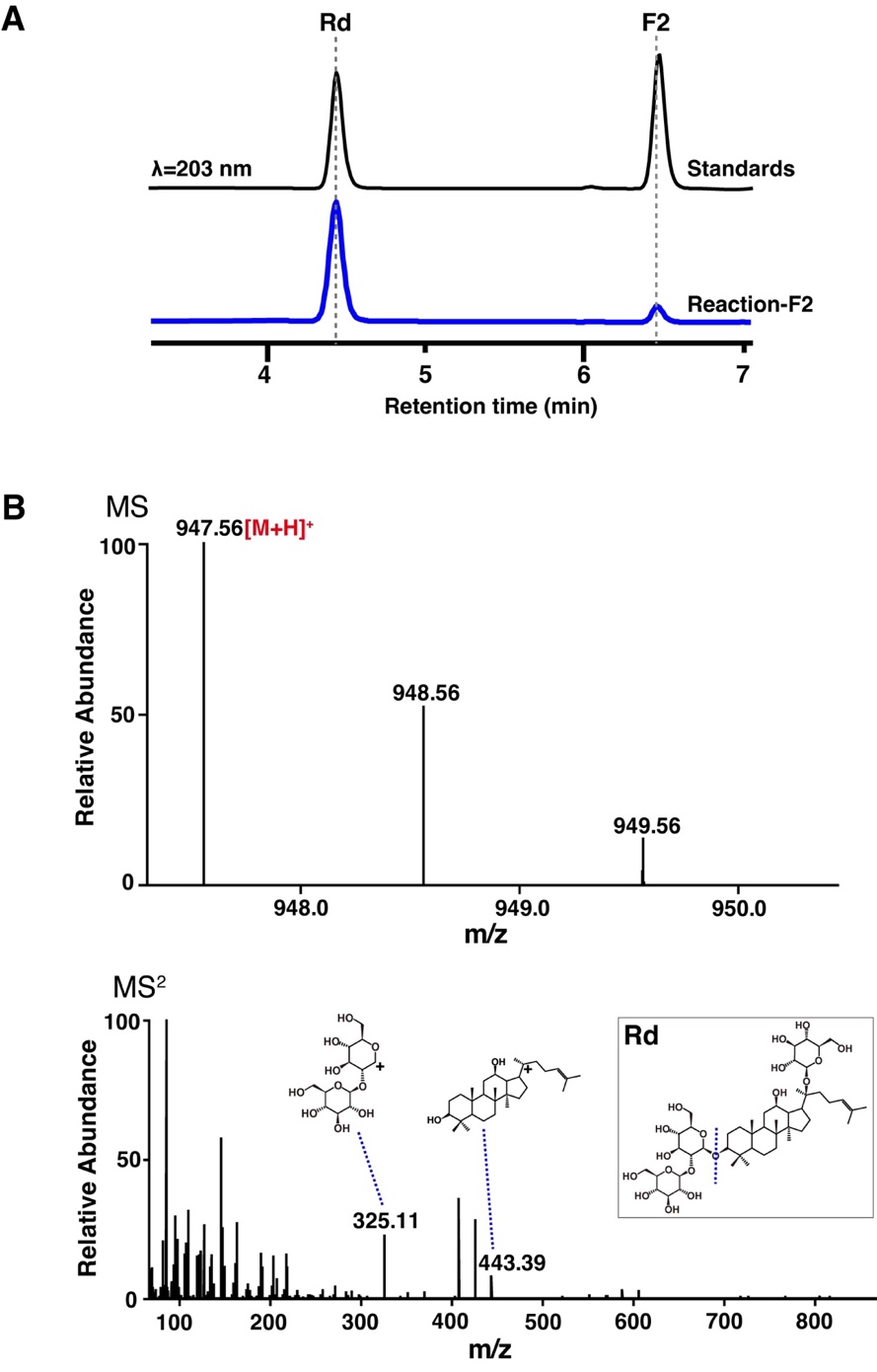
**

**Figure S12. Pq3-O-UGT2 catalyzes glycosylation of Ginsenoside F2 to generate Ginsenoside Rd.** **A,** HPLC results of the reaction mixtures of F2 glycosylation by Pq3-O-UGT2 and **B,** mass spectra of the product.

**
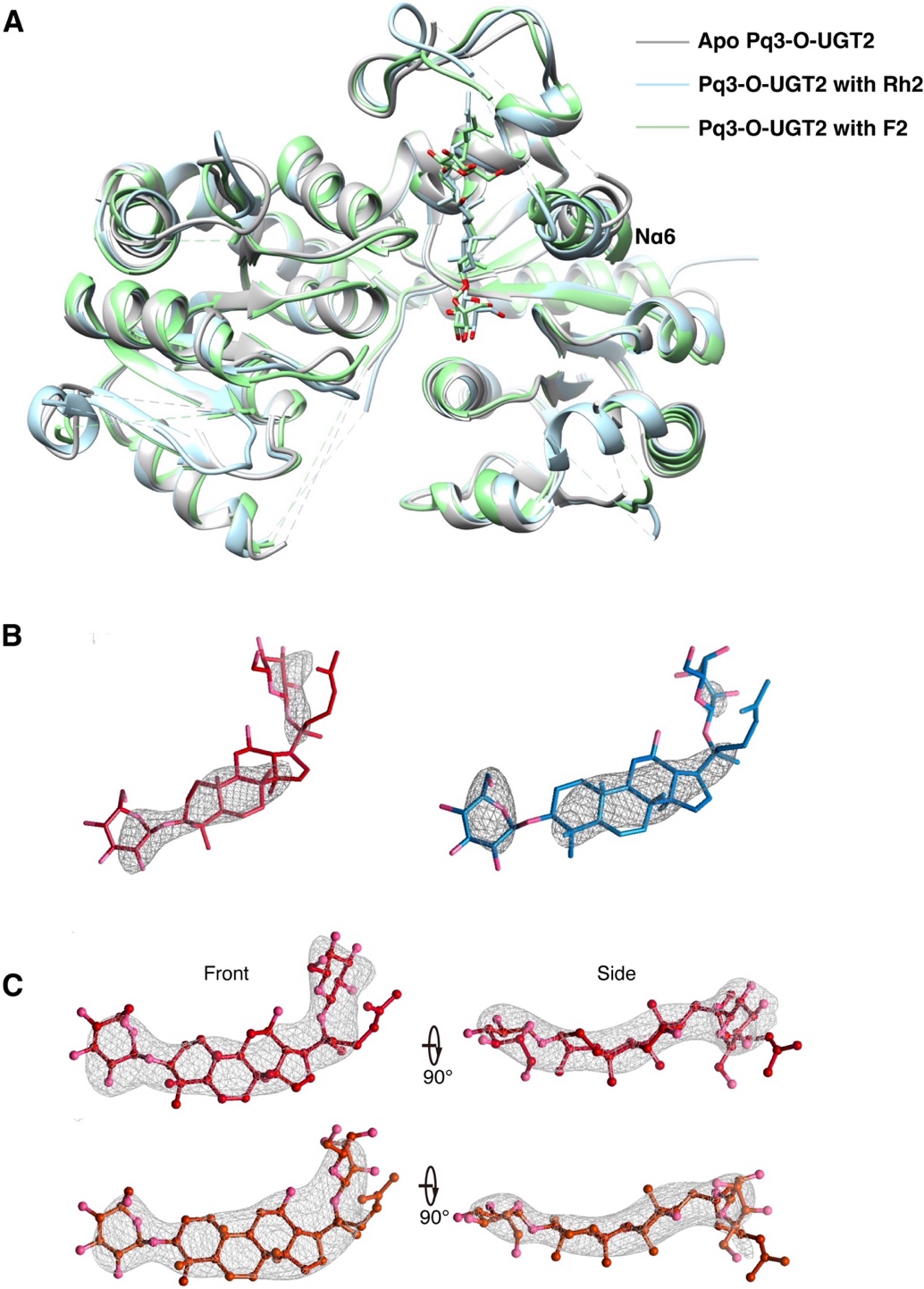
**

**Figure S13. Crystal structure of Pq3-O-UGT2 in complex with F2. A,** Structure alignment of apo Pq3-O-UGT2 and its complex with Rh2 and F2. Rh2 and F2 are shown with sticks. **B,** The experimental Fo-Fc omit maps (contoured at 2.5σ) for F2 in the two protomers of the complex structure of Pq3-O-UGT2 and F2. **C**, The final refined 2Fo-Fc electron density maps (contoured at 1.5σ) for F2 in the two protomers of the complex structure.

**
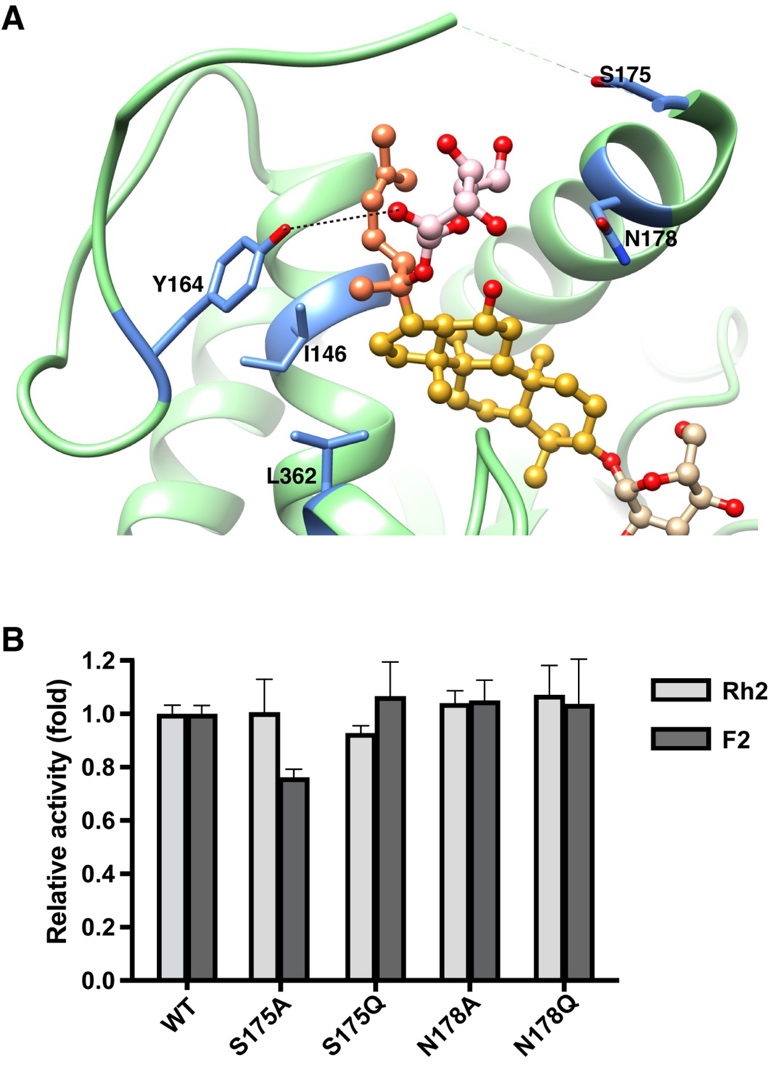
**

**Figure S14. Ser175 specifically contributes to F2 recognition by Pq3-O-UGT2. A,** Residues around the alky tail and C20-glucose of F2. Key residues are labeled and highlighted as sticks. F2 is presented as a stick-and-ball model, with the C3-glucose colored in tan, the tetracyclic ring in gold, alky tail in coral, C20-glucose in pink, and oxygen atoms in red. **B,** Relative activities of Pq3-O-UGT2 mutants towards F2. Error bars represent standard deviation from three repeats.


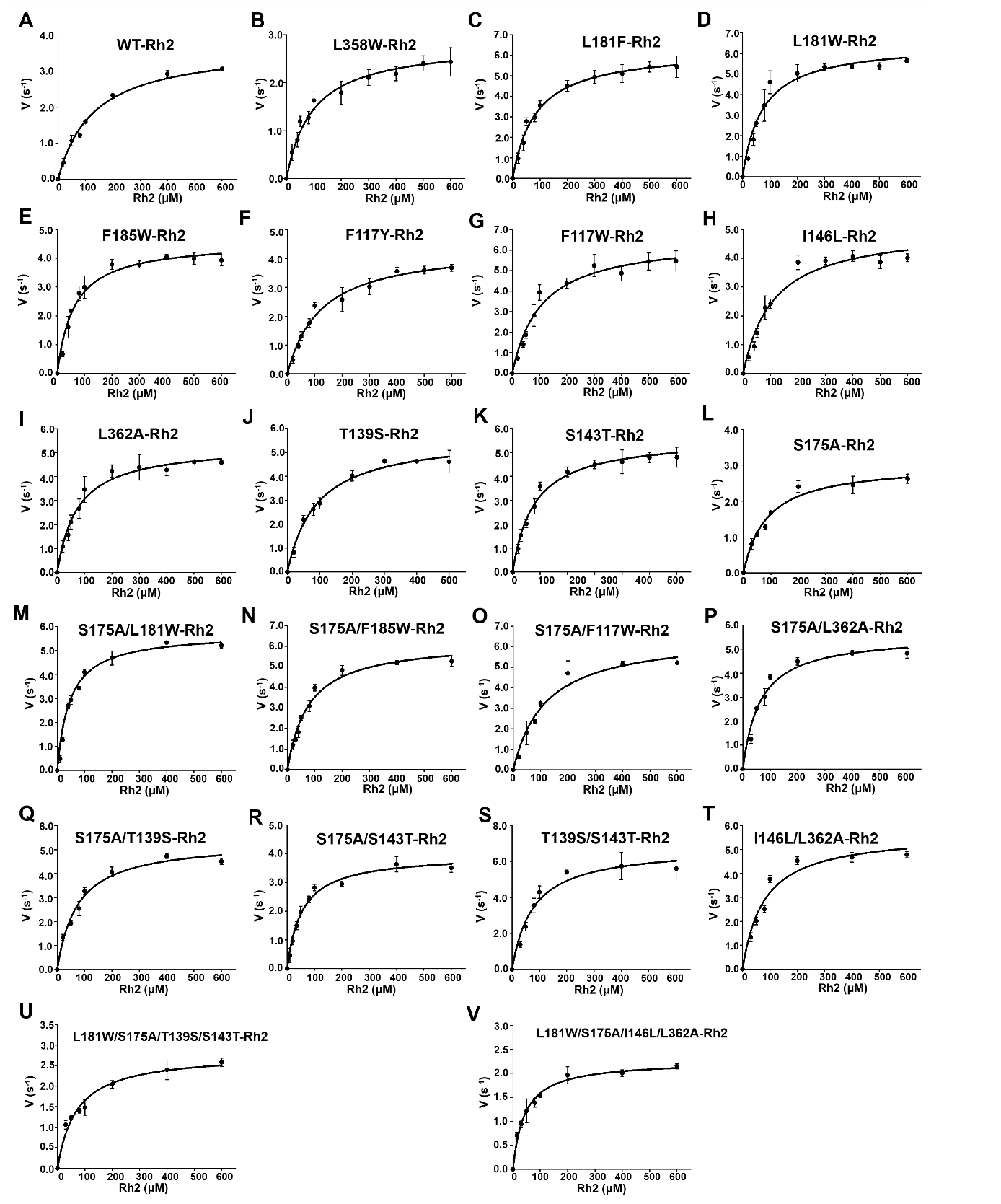


**Figure S15. Kinetic parameter determination for recombinant Pq3-O-UGT2 and its mutants using Rh2 as the acceptor and UDP-Glc as the donor.**


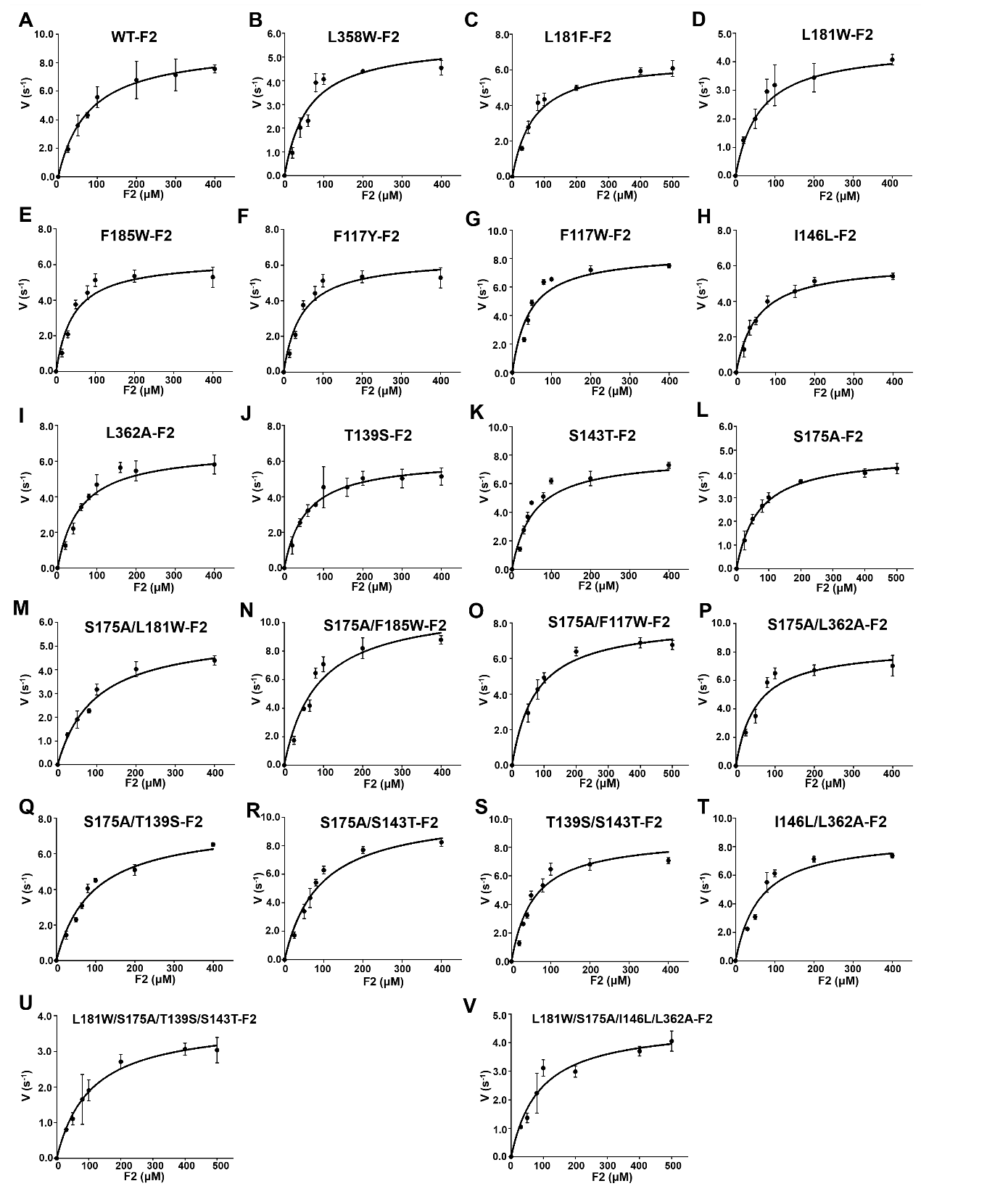


**Figure S16. Kinetic parameter determination for recombinant Pq3-O-UGT2 and its mutants using F2 as the acceptor and UDP-Glc as the donor.**

**
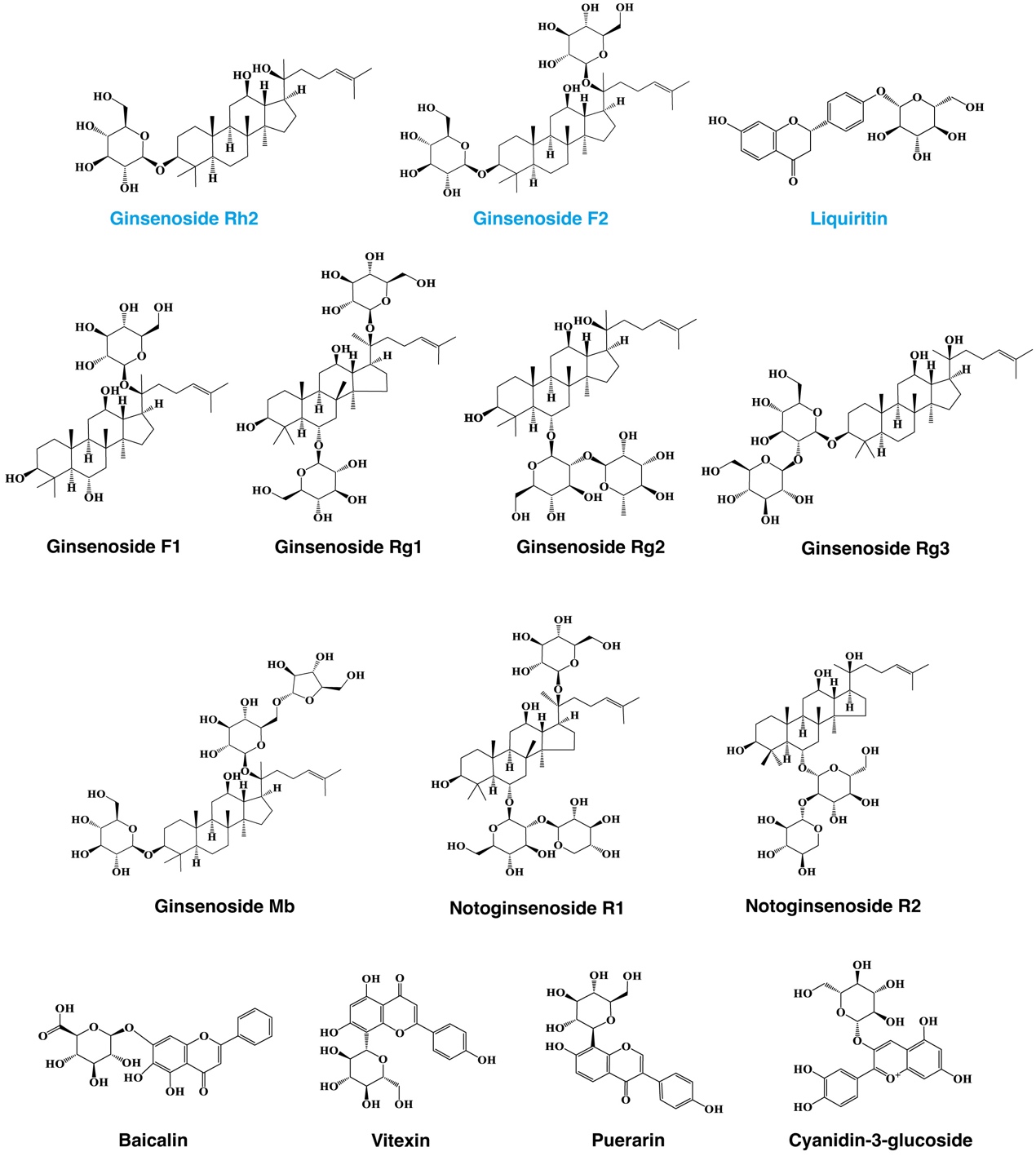
**

**Figure S17. Molecular structures of compounds in the library.** Compounds confirmed to be Pq3-O-UGT2 substrates are indicated in blue.


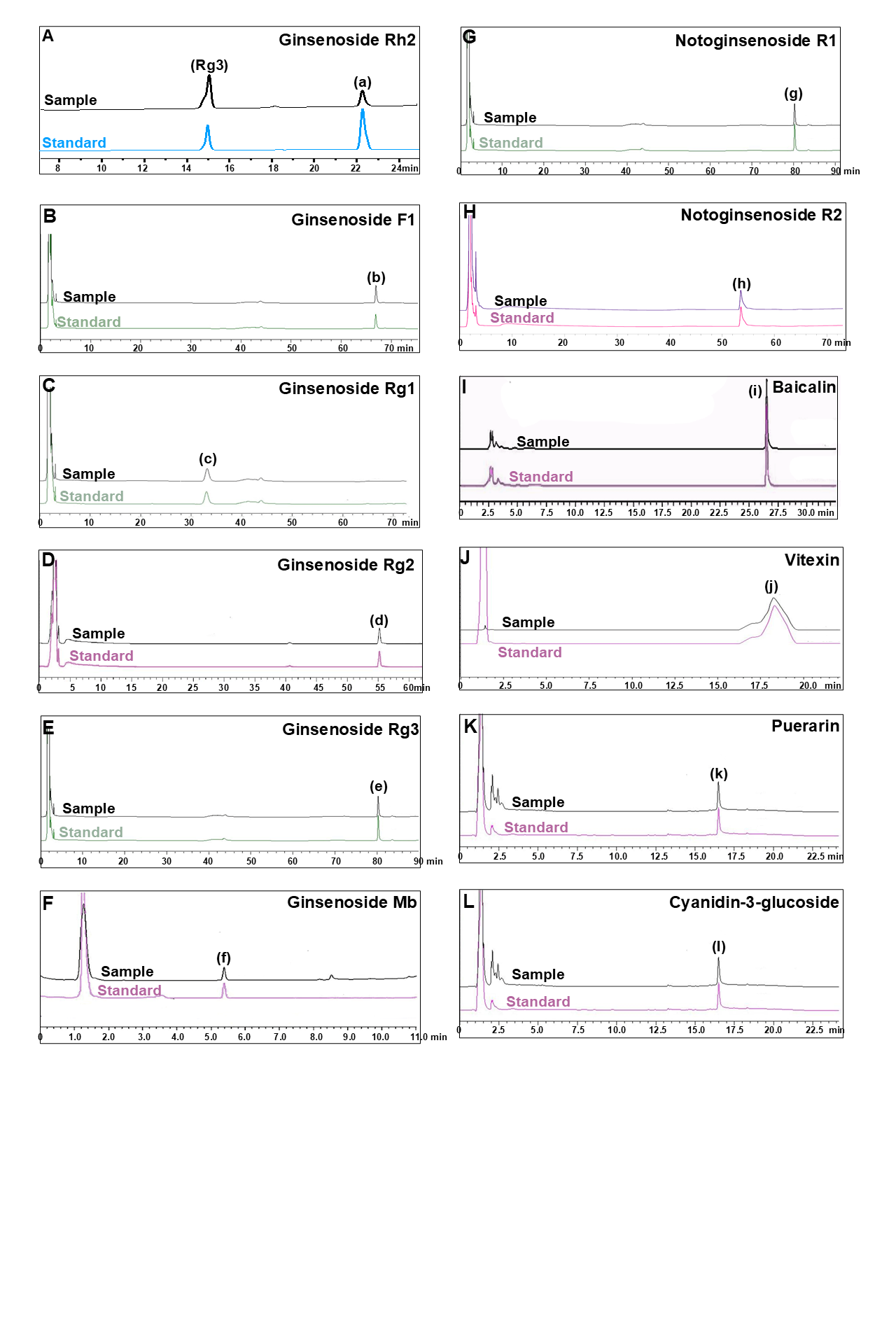


**Figure S18. HPLC profiles of reaction mixtures catalyzed by Pq3-O-UGT2.** The sugar acceptors used in these reactions are listed in Figure S17. Reaction mixtures without the enzyme served as controls (standard) to identify the peaks corresponding to each sugar acceptor. The peaks of the sugar acceptors are indicated in the profiles. Results for Ginsenoside F2 and Liquiritin are shown in Figure S12A and Figure 7A, respectively.

**
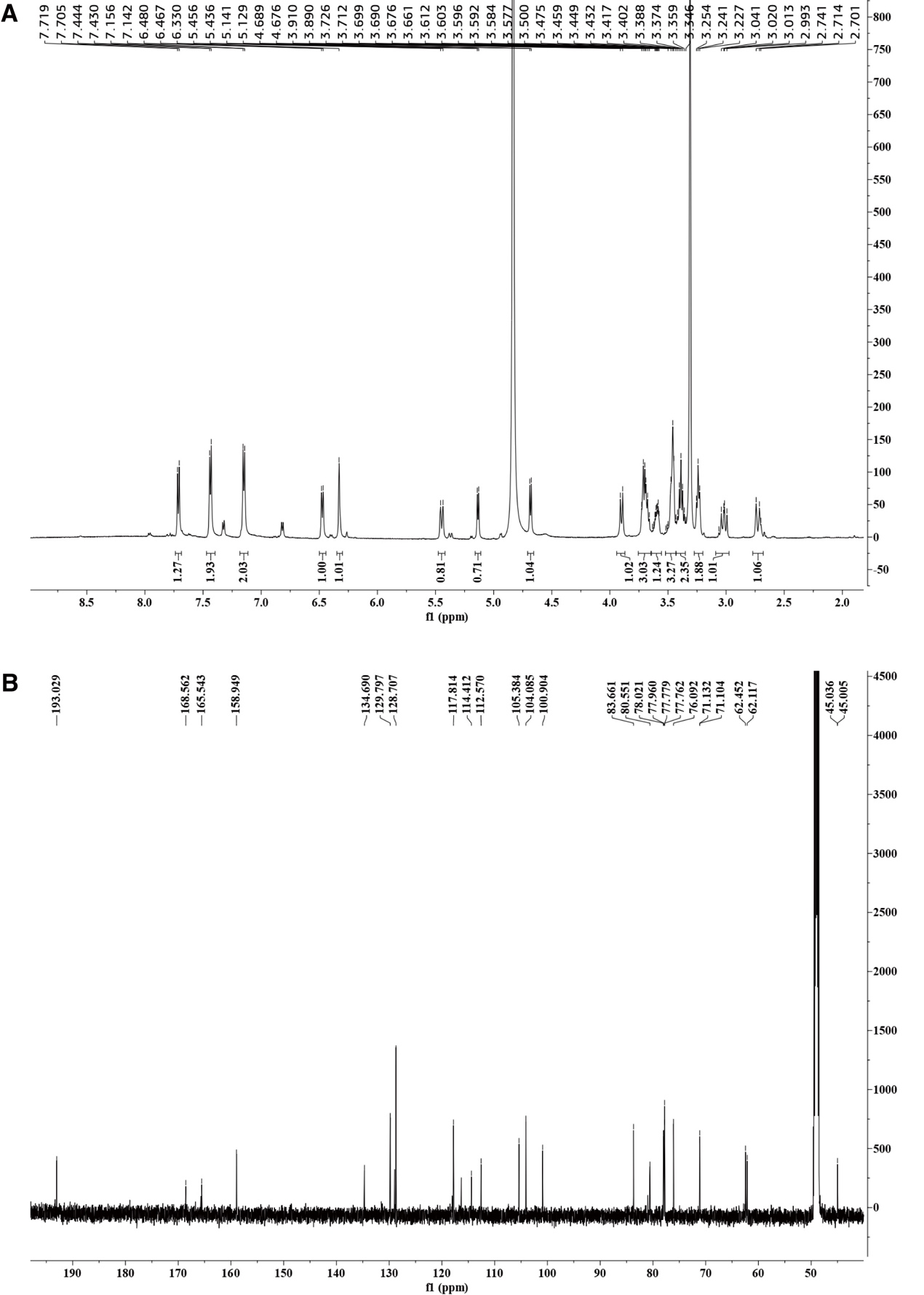
**

**Figure S19. NMR spectrum of** **the glycosylated product of liquiritin by Pq3-O-UGT2.**


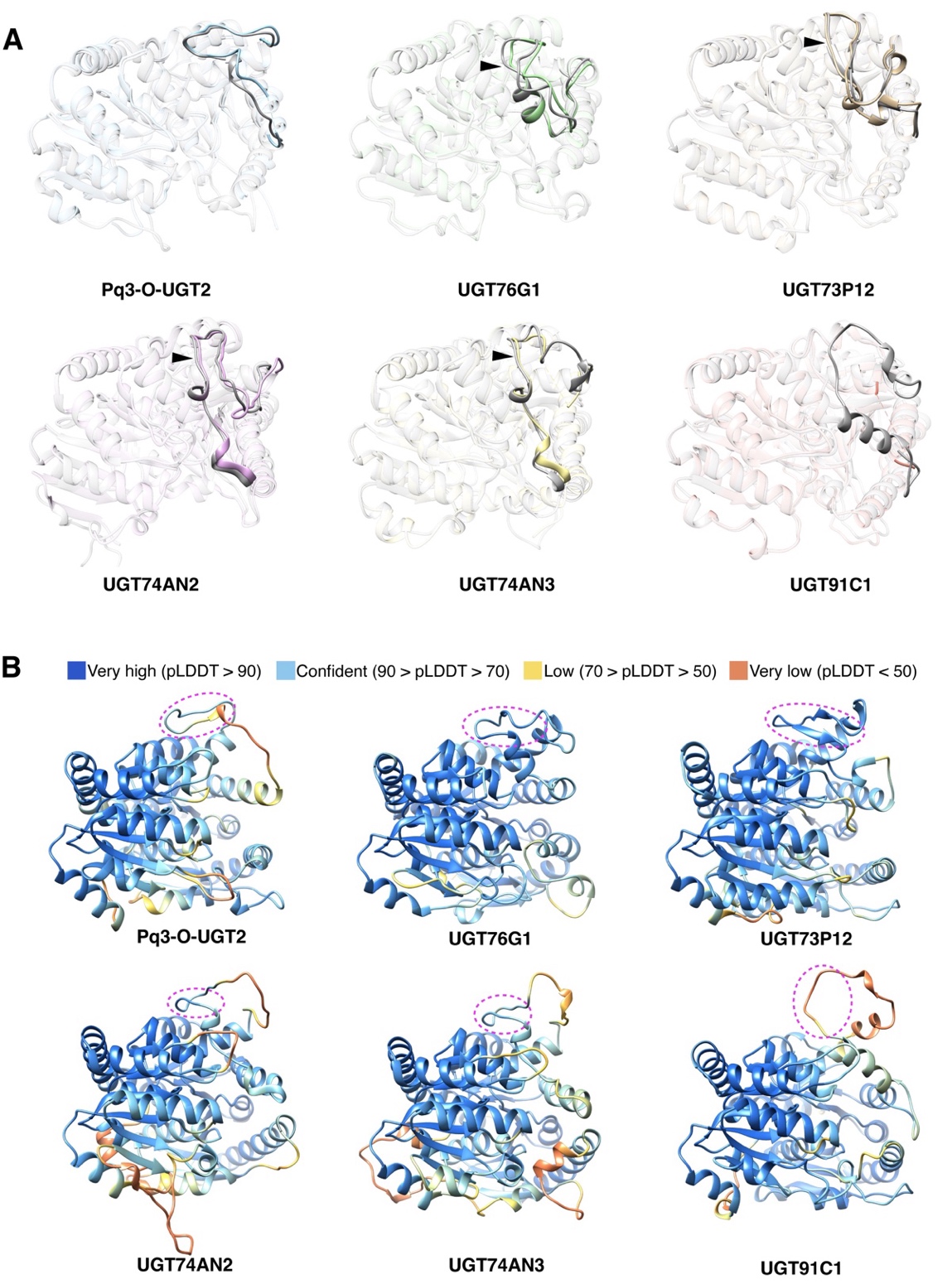


**Figure S20. Analysis of the acceptor binding pocket of UGTs with predicted structures. A,** Alignment of crystal structures and predicted structures of plant UGTs catalyzing large and extended acceptors. The predicted structures were generated by AlphaFold2. All predicted structures are shown in gray. The Nα5-Nα6 linker is highlighted with the remaining structures depicted in semitransparency. The hairpin in the Nα5-Nα6 linker is indicated with arrow heads. **B,** Confidence of the predicted structures. The predicted structures of Pq3-O-UGT2, UGT76G1, UGT73P12, UGT74AN2, UGT74AN3, and UGT91C1 are color-coded according to prediction confidence. The Nα5-Nα6 turn and Nα5-Nα6 hairpin are indicated with magenta circles.

**
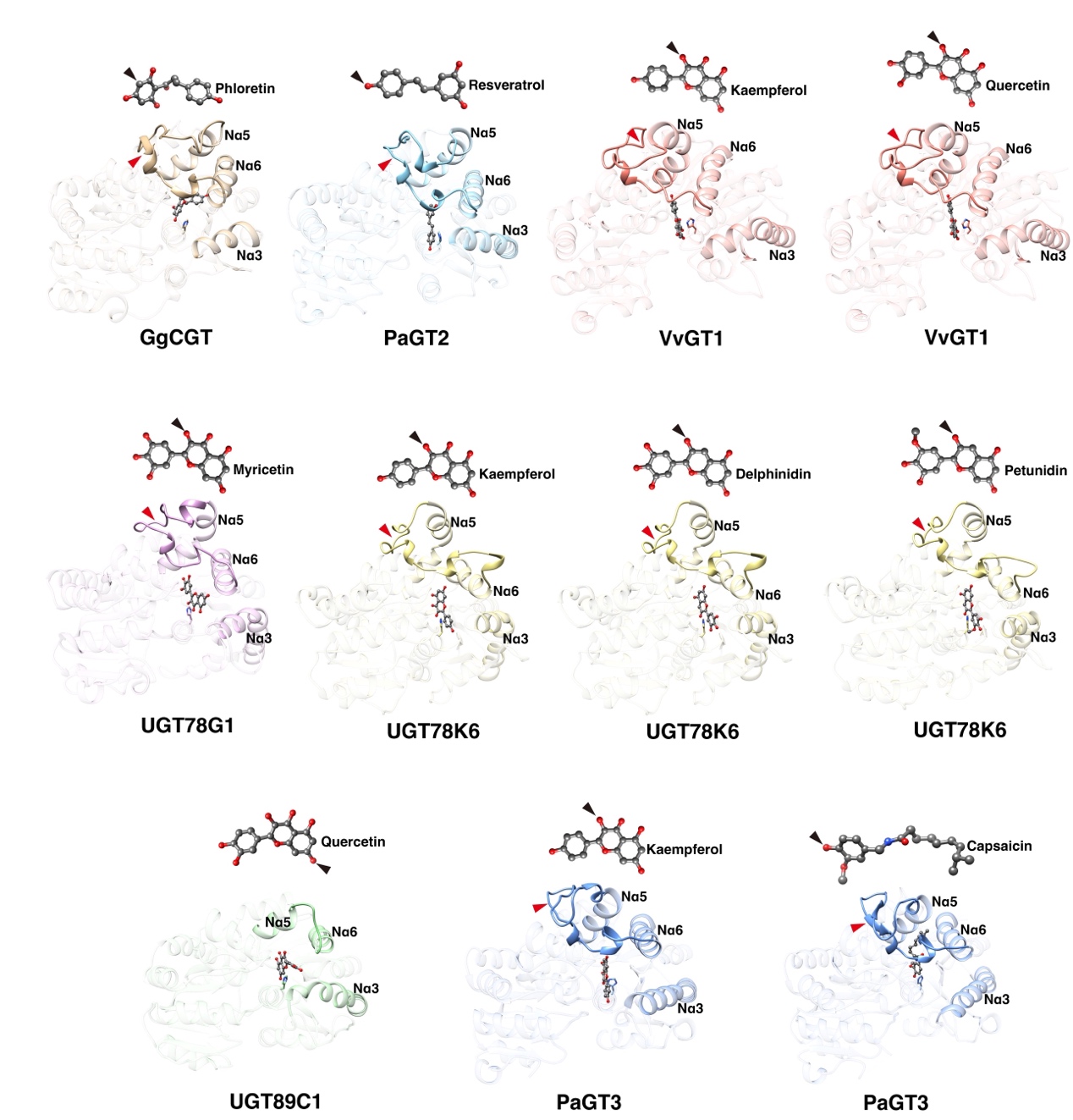
**

**Figure S21. Binding orientations of** **polyphenols to plant UGTs.** The structures of the acceptors are depicted above each complex, highlighting the group to be glycosylated with black arrowheads. The Nα3, Nα5, Nα6 helices, and the Nα5-Nα6 linker are emphasized in the complex structures, with other regions presented in semitransparency. The hairpin in the Nα5-Nα6 linker is indicated by red arrowheads.

**Table S1. Data collection and refinement statistics.**

|  | Pq3-O-UGT2  (8JZQ) | Pq3-O-UGT2-Rh2 (8K08) | Pq3-O-UGT2-F2  (8K09) |
| --- | --- | --- | --- |
| Data collection | | | |
| Beamline | BL18U1, SSRF | BL19U1, SSRF | BL10U2, SSRF |
| Wave length (Å) | 0.97853 | 0.97853 | 0.97918 |
| Space group | *P*1 | *P*2_1_2_1_2_1_ | *P*2_1_2_1_2_1_ |
| *a, b, c* (Å) | 47.0,80.9,126.4 | 70.4,90.2,155.9 | 60.3,88.2,164.5 |
| *α, β, γ* (°) | 94.0,90.0,93.8 | 90.0, 90.0, 90.0 | 90.0, 90.0, 90.0 |
| Resolution (Å) * | 50.00-2.90  (2.95-2.90) | 52.22-3.50  (3.625-3.50) | 49.76-3.36  (3.45-3.36) |
| Unique reflections* | 39,238 (2,010) | 12,280 (1,257) | 13,100 (947) |
| Completeness (%) * | 95.0 (95.8) | 93.78 (99.60) | 99.9 (100.0) |
| Multiplicity | 2.8 (2.9) | 7.4 (7.7) | 11.9 (10.4) |
| *I* / σ*I** | 9.3 (2.3) | 15.04 (1.88) | 12.2 (1.7) |
| R_merge_* | 0.13(0.75) | 0.1081 (1.25) | 0.126 (1.454) |
| CC _1/2_* | 0.992(0.543) | 0.999 (0.569) | 0.998 (0.528) |
| **Refinement** | | | |
| Resolution (Å) * | 31.36-2.90  (2.99-2.90) | 52.22-3.50  (3.625-3.50) | 36.85-3.36  (3.48-3.36) |
| Reflections used in refinement | 39,084 (3,826) | 12,245 (1,257) | 13,049 (1,264) |
| Reflections used for R-free | 1,922 (176) | 573 (57) | 651 (74) |
| *R*work/ *R*free (%)* | 30.4(36.8)/ 33.2(42.8) | 29.3(41.6)/ 31.8(44.2) | 29.7(38.0)/  33.4 (38.9) |
| No. non-hydrogen atoms | | | |
| Protein | 11872 | 6241 | 5993 |
| Ligand/ion | - | 88 | 110 |
| Average *B*-factor (Å^2^) | | | |
| Protein | 70.4 | 122.1 | 138.4 |
| Ligand/ion | - | 104.8 | 115.2 |
| R.m.s deviations | | | |
| Bond lengths (Å) | 0.008 | 0.003 | 0.005 |
| Bond angles (°) | 1.18 | 0.63 | 0.83 |
| Ramachandran Plot | | | |
| Favored (%) | 91.6 | 91.5 | 90.2 |
| Allowed (%) | 8.4 | 8.5 | 9.8 |
| Outliers (%) | 0 | 0 | 0 |

*Values in parentheses are for highest-resolution shell.

**Table S2**. **Apparent kinetic parameters of Pq3-O-UGT2 and its mutants towards Rh2 and F2.**

| Pq3-O-UGT2 | Substrate | *K_M_*,  μM | *k*_cat_,  s^-1^ | *k*_cat_/*K_M_*,  s^-1^·mΜ^-1^ | Fold to WT |
| --- | --- | --- | --- | --- | --- |
| WT | Rh2 | 130.02±25.81 | 3.71±0.31 | 28.54±2.41 | 1 |
|  | F2 | 77.69±8.46 | 9.16±0.34 | 117.94±4.34 | 1 |
| L358W | Rh2 | 92.05±30.97 | 2.85±0.34 | 30.92±11.08 | 1.08 |
|  | F2 | 60.37±19.85 | 5.65±0.66 | 93.57±10.86 | 0.79 |
| L181F | Rh2 | 83.87±11.53 | 6.30±0.31 | 75.08±3.75 | 2.63 |
|  | F2 | 80.26±0.49 | 5.68±0.27 | 70.74±3.34 | 0.60 |
| L181W | Rh2 | 72.17±10.49 | 6.51±0.26 | 90.17±3.61 | 3.15 |
|  | F2 | 83.61±8.43 | 5.04±0.23 | 60.29±2.75 | 0.51 |
| F185W | Rh2 | 61.45±8.87 | 4.59±0.18 | 74.74±2.93 | 2.62 |
|  | F2 | 51.24±14.13 | 6.33±0.74 | 123.45±14.42 | 1.05 |
| F117Y | Rh2 | 122.90±15.79 | 4.46±0.20 | 36.30±1.59 | 1.27 |
|  | F2 | 45.53±11.63 | 6.45±0.53 | 141.66±11.65 | 1.20 |
| F117W | Rh2 | 105.41±18.59 | 6.60±0.38 | 62.63±3.57 | 2.14 |
|  | F2 | 43.57±12.68 | 8.42±0.67 | 193.19±15.28 | 1.64 |
| I146L | Rh2 | 109.57±21.81 | 5.07±0.33 | 46.26±3.01 | 1.62 |
|  | F2 | 53.54±9.79 | 6.16±0.33 | 115.05±6.21 | 0.98 |
| L362A | Rh2 | 73.73±9.63 | 5.32±0.20 | 72.20±2.72 | 2.53 |
|  | F2 | 57.00±19.47 | 6.67±0.73 | 116.96±12.85 | 0.99 |
| T139S | Rh2 | 91.05±10.49 | 5.70±0.21 | 62.66±2.3 | 2.20 |
|  | F2 | 54.49±6.38 | 6.13±0.22 | 112.59±3.99 | 0.95 |
| S143T | Rh2 | 78.20±8.87 | 5.63±0.20 | 72.02±2.59 | 2.52 |
|  | F2 | 54.53±10.87 | 7.91±0.61 | 145.15±11.28 | 1.23 |
| S175A | Rh2 | 90.83±14.35 | 2.65±0.14 | 29.19±1.53 | 1.02 |
|  | F2 | 80.52±12.23 | 5.01±0.27 | 62.22±3.40 | 0.53 |
| S175A/L181W | Rh2 | 45.43±3.61 | 5.74±0.13 | 126.40±2.93 | 4.43 |
|  | F2 | 99.98±13.00 | 5.43±0.32 | 54.29±2.05 | 0.46 |
| S175A/F185W | Rh2 | 77.63±10.18 | 6.27±0.29 | 80.76±3.72 | 2.83 |
|  | F2 | 83.77±7.23 | 10.84±1.02 | 129.45±12.20 | 1.10 |
| S175A/F117W | Rh2 | 119.28±23.88 | 6.63±0.47 | 55.60±3.93 | 1.95 |
|  | F2 | 73.90±12.79 | 8.12±0.42 | 109.92±5.70 | 0.93 |
| S175A/L362A | Rh2 | 62.73±11.77 | 5.59±0.31 | 89.17±4.99 | 3.12 |
|  | F2 | 55.38±15.74 | 10.10±1.00 | 152.02±14.98 | 1.29 |
| S175A/T139S | Rh2 | 74.02±12.48 | 5.36±0.28 | 72.36±3.78 | 2.54 |
|  | F2 | 90.53±17.27 | 7.68±1.28 | 84.81±14.11 | 0.72 |
| S175A/S143T | Rh2 | 54.32±6.11 | 3.97±0.14 | 73.12±2.53 | 2.56 |
|  | F2 | 90.37±17.11 | 10.04±0.84 | 111.04±9.249 | 0.94 |
| T139S/S143T | Rh2 | 76.38±16.02 | 6.82±0.46 | 89.26±5.98 | 3.13 |
|  | F2 | 55.66±13.13 | 10.50±0.90 | 156.92±13.46 | 1.33 |
| I146L/L362A | Rh2 | 78.28±19.48 | 5.69±0.46 | 72.70±5.82 | 2.55 |
|  | F2 | 61.79±15.93 | 8.71±0.67 | 141.00±10.89 | 1.20 |
| L181W/S175A/ T139S/S143T | Rh2 | 69.32±12.02 | 2.80±0.15 | 40.32±2.16 | 1.41 |
|  | F2 | 104.98±12.01 | 3.83±0.16 | 36.51±1.50 | 0.31 |
| L181W/S175A/ I146L/L362A | Rh2 | 42.41±4.29 | 2.26±0.06 | 53.40±1.47 | 1.87 |
|  | F2 | 87.61±22.15 | 4.65±0.40 | 53.10±4.54 | 0.45 |

**Table S3**. **Primers used for mutagenesis in this study.**

| F15A-F | TTGCTACCAGCGTTAGCCCATGGTCACATATCTCCCTTCTTTGA |
| --- | --- |
| F15A-R | TTGCGAGTTGTTTGGCTAGCTCAAAGAAGGGAGATATGTGACCAT |
| H20A-F | TTTTTAGCCCATGGTGCCATATCTCCCTTCTTTGA |
| H20A-R | TCAAAGAAGGGAGATATGGTACCATGGGCTAAAAA |
| H20N-F | ATTTTTAGCCCATGGTAACATATCTCCCTTCT |
| H20N-R | AGAAGGGAGATATGTTACCATGGGCTAAAAAT |
| D116A-F | ACCCCGATTTGCTTATTTATGCGTTCAATCCCTCATGGGCA |
| D116A-R | TGCCCATGAGGGATTGAACGCATAAATAAGCAAATCGGGGT |
| D116N-F | CCGATTTGCTTATTTATAACTTCAATCCCTCATGGGCA |
| D116N-R | TGCCCATGAGGGATTGAAGTTATAAATAAGCAAATCGG |
| F117A-F | ATTTGCTTATTTATGATGCCAATCCCTCATGGGCACCGGA |
| F117A-R | TCCGGTGCCCATGAGGGATTGGCATCATAAATAAGCAAATCGGGGT |
| F117L-F | TTGCTTATTTATGATCTGAATCCCTCATGGGCA |
| F117L-R | TGCCCATGAGGGATTCAGATCATAAATAAGCAA |
| F117Y-F | TTGCTTATTTATGATTATAATCCCTCATGGGCA |
| F117Y-R | TGCCCATGAGGGATTATAATCATAAATAAGCAA |
| F117W-F | TTGCTTATTTATGATTGGAATCCCTCATGGGCA |
| F117W-R | TGCCCATGAGGGATTCCAATCATAAATAAGCAA |
| N118A-F | ATTTATGATTTCGCGCCCTCATGGGCACCGGA |
| N118A-R | TGCCCATGAGGGCGCGAAATCATAAATAAGCAAAT |
| T139A-F | TTTATTTCCTAACCGCGGCAGCAGCCAGCTCTT |
| T139A-R | AAGAGCTGGCTGCTGCCGCGGTTAGGAAATAAACTGC |
| T139S-F | AGTTTATTTCCTAACCAGCGCAGCAGCCAGCTCT |
| T139S-R | AGAGCTGGCTGCTGCGCTGGTTAGGAAATAAACT |
| T139N-F | AGTTTATTTCCTAACCAACGCAGCAGCCAGCTCT |
| T139N-R | AGAGCTGGCTGCTGCGTTGGTTAGGAAATAAACT |
| S143A-F | ACCACGGCAGCAGCCGCGTCTTCCATTGGCCTA |
| S143A-R | TAGGCCAATGGAAGACGCGGCTGCTGCCGTGGT |
| S143T-F | ACCACGGCAGCAGCCACCTCTTCCATTGGCCTA |
| S143T-R | TAGGCCAATGGAAGAGGTGGCTGCTGCCGTGGT |
| I146A-F | AGCAGCCAGCTCTTCCGCGGGCCTACATGCTTTCAAA |
| I146A-R | TTTGAAAGCATGTAGGCCCGCGGAAGAGCTGGCTGCTGCCGT |
| I146L-F | AGCAGCCAGCTCTTCCCTGGGCCTACATGCTTTCAA |
| I146L-R | TTGAAAGCATGTAGGCCCAGGGAAGAGCTGGCTGCT |
| I146F-F | AGCAGCCAGCTCTTCCTTTGGCCTACATGCTTTCAA |
| I146F-R | TTGAAAGCATGTAGGCCAAAGGAAGAGCTGGCTGCT |
| G147L-F | AGCCAGCTCTTCCATTCTGCTACATGCTTTCAA |
| G147L-R | TTGAAAGCATGTAGCAGAATGGAAGAGCTGGCT |
| G147F-F | AGCCAGCTCTTCCATTTTTCTACATGCTTTCAA |
| G147F-R | TGAAAGCATGTAGAAAAATGGAAGAGCTGGC |
| F163Y-F | ACCCATTTCCAGATTATTATGATAACAGT |
| F163Y-R | ACTGTTATCATAATAATCTGGAAATGGGT |
| F163W-F | ACCCATTTCCAGATTGGTATGATAACAGT |
| F163W-R | ACTGTTATCATACCAATCTGGAAATGGGT |
| F163D-F | ACCCATTTCCAGATGATTATGATAACAGTAATATTATCC |
| F163D-R | ACTGTTATCATAATCATCTGGAAATGGGTATT |
| Y164A-F | TCCAGATTTTGCGGATAACAGTAATATTA |
| Y164A-R | ACTGTTATCCGCAAAATCTGGAAATGGGT |
| Y164L-F | TCCAGATTTTCTGGATAACAGTAATATTA |
| Y164L-R | ACTGTTATCCAGAAAATCTGGAAATGGGT |
| Y164F-F | TCCAGATTTTTTTGATAACAGTAATATTA |
| Y164F-R | ACTGTTATCAAAAAAATCTGGAAATGGGT |
| Y164W-F | TCCAGATTTTTGGGATAACAGTAATATTA |
| Y164W-R | ACTGTTATCCCAAAAATCTGGAAATGGGT |
| S175A-F | ATCCCTGAACCTCCTGCGGCAGATAACATGAA |
| S175A-R | TTCATGTTATCTGCCGCAGGAGGTTCAGGGAT |
| S175Q-F | ATCCCTGAACCTCCTCAGGCAGATAACATGAA |
| S175Q-R | TTCATGTTATCTGCCTGAGGAGGTTCAGGGAT |
| N178A-F | ACCTCCTTCTGCAGATGCGATGAAGCTACTTCAT |
| N178A-R | ATGAAGTAGCTTCATCGCATCTGCAGAAGGAGGT |
| N178Q-F | ACCTCCTTCTGCAGATCAGATGAAGCTACTTCAT |
| N178Q-R | ATGAAGTAGCTTCATCTGATCTGCAGAAGGAGGT |
| L181A-F | TTCGAAACAAGCGATCCAATCATGAAGTAGCTT |
| L181A-R | ATAAAATCATGAAGCGCCTTCATGTTATCTGCA |
| L181F-F | TGCAGATAACATGAAGTTTCTTCATGATTTTAT |
| L181F-R | ATAAAATCATGAAGAAACTTCATGTTATCTGCA |
| L181W-F | TGCAGATAACATGAAGTGGCTTCATGATTTTAT |
| L181W-R | ATAAAATCATGAAGCCACTTCATGTTATCTGCA |
| F185A-F | ATGAAGCTACTTCATGATGCGATCGCTTGTTTCGAACGAT |
| F185A-R | ATCGTTCGAAACAAGCGATCGCATCATGAAGTAGCTTCATGT |
| F185L-F | AAGCTACTTCATGATCTGATCGCTTGTTTCGAA |
| F185L-R | TTCGAAACAAGCGATCAGATCATGAAGTAGCTT |
| F185W-F | AAGCTACTTCATGATTGGATCGCTTGTTTCGAA |
| F185W-R | TTCGAAACAAGCGATCCAATCATGAAGTAGCTT |
| L358A-F | ATTGCCATGGCCAGGCATGCGGATCAGCCTTTGAATGGTA |
| L358A-R | ACCATTCAAAGGCTGATCCGCATGCCTGGCCATGGCAAT |
| L358F-F | TGCCATGGCCAGGCATTTTGATCAGCCTTTGAAT |
| L358F-R | ATTCAAAGGCTGATCAAAATGCCTGGCCATGGCA |
| L358W-F | TGCCATGGCCAGGCATTGGGATCAGCCTTTGAAT |
| L358W-R | ATTCAAAGGCTGATCCCAATGCCTGGCCATGGCA |
| D359A-F | ATGGCCAGGCATCTTGCGCAGCCTTTGAATGGT |
| D359A-R | TACCATTCAAAGGCTGCGCAAGATGCCTGGCCAT |
| D359N-F | TGGCCAGGCATCTCAACCAGCCTTTGAATGG |
| D359N-R | CCATTCAAAGGCTGGTTGAGATGCCTGGCCA |
| Q360A-F | TGGCCAGGCATCTCGATGCGCCTTTGAATGGTAAGCT |
| Q360A-R | AGCTTACCATTCAAAGGCGCATCGAGATGCCTGGCCA |
| Q360E-F | TGGCCAGGCATCTCGATGAACCTTTGAATGGTAAGCT |
| Q360E-R | AGCTTACCATTCAAAGGTTCATCGAGATGCCTGGCCA |
| D359NQ360E-F | TGCCATGGCCAGGCATCTCAACGAACCTTTGAATGGTAAGCTGGC |
| D359NQ360E-R | GCCAGCTTACCATTCAAAGGTTCGTTGAGATGCCTGGCCATGGCA |
| L362A-F | ATCTCGATCAGCCTGCGAATGGTAAGCT |
| L362A-R | AGCTTACCATTCGCAGGCTGATCGAGAT |
| L362F-F | ATCTCGATCAGCCTTTTAATGGTAAGCT |
| L362F-R | AGCTTACCATTAAAAGGCTGATCGAGAT |
| L181WF185W-F | AAGTGGCTTCATGATTGGATCGCTTGTTTCGAACGATCT |
| L181WF185W-R | AAGCGATCCAATCATGAAGCCACTTCATGTTATCTGCAGAA |
| T139SS143T-F | ACCAGCGCAGCAGCCACCTCTTCCATTGGCCTACAT |
| T139SS143T-R | TGGAAGAGGTGGCTGCTGCGCTGGTTAGGAAATAAACTGCC |
| S175A/L181W-F | TCCTGCGGCAGATAACATGAAGTGGCTTCATGATTTTATCGCT |
| S175A/L181W-R | TTCATGTTATCTGCCGCAGGAGGTTCA |
| S175A/F185W-F | TCCTGCGGCAGATAACATGAAGCTACTTCATGATTGGATCGCTTGTTTCGAACGATCTT |
| S175A/F185W-R | TTCATGTTATCTGCCGCAGGAGGTTCAGGGA |
